# Sequence-controlled RNA self-processing: computational design, biochemical analysis, and visualization by AFM

**DOI:** 10.1261/rna.047670.114

**Published:** 2015-07

**Authors:** Sonja Petkovic, Stefan Badelt, Stephan Block, Christoph Flamm, Mihaela Delcea, Ivo Hofacker, Sabine Müller

**Affiliations:** 1 Institute for Biochemistry, Ernst-Moritz-Arndt University Greifswald, 17487 Greifswald, Germany; 2 Institute for Theoretical Chemistry, University of Vienna, A-1090 Vienna, Austria; 3 ZIK HIKE—Center for Innovation Competence, Humoral Immune Reactions in Cardiovascular Diseases, Ernst-Moritz-Arndt University Greifswald, 17489 Greifswald, Germany; 4 Research Group Bioinformatics and Computational Biology, University of Vienna, A-1090 Vienna, Austria

**Keywords:** AFM, circularization, computational design, hairpin ribozyme, RNA, self-processing

## Abstract

Reversible chemistry allowing for assembly and disassembly of molecular entities is important for biological self-organization. Thus, ribozymes that support both cleavage and formation of phosphodiester bonds may have contributed to the emergence of functional diversity and increasing complexity of regulatory RNAs in early life. We have previously engineered a variant of the hairpin ribozyme that shows how ribozymes may have circularized or extended their own length by forming concatemers. Using the Vienna RNA package, we now optimized this hairpin ribozyme variant and selected four different RNA sequences that were expected to circularize more efficiently or form longer concatemers upon transcription. (Two-dimensional) PAGE analysis confirms that (i) all four selected ribozymes are catalytically active and (ii) high yields of cyclic species are obtained. AFM imaging in combination with RNA structure prediction enabled us to calculate the distributions of monomers and self-concatenated dimers and trimers. Our results show that computationally optimized molecules do form reasonable amounts of trimers, which has not been observed for the original system so far, and we demonstrate that the combination of theoretical prediction, biochemical and physical analysis is a promising approach toward accurate prediction of ribozyme behavior and design of ribozymes with predefined functions.

## INTRODUCTION

RNA processing plays a fundamental role in the cellular life cycle. RNA molecules are permanently synthesized, modified, edited, truncated, or abolished. In viruses, viroids, and satellite RNAs with circular RNA genomes, replication follows a rolling circle mechanism, thus initially producing linear concatemeric versions of the RNA genome (Flores et al. 2011). Further processing is required to convert the concatemers back to monomers that subsequently are cyclized to yield the final replication product: a cyclic RNA complementary to the template. This processing is dependent on specific RNA structural motifs that support reaction at the site of cleavage and ligation (Hampel and Tritz 1989; DeYoung et al. 1995). Among those, the hammerhead and the hairpin ribozyme are probably the best studied small RNAs with catalytic activity (Hammann et al. 2012; Müller et al. 2012).

**SCHEME 1.**
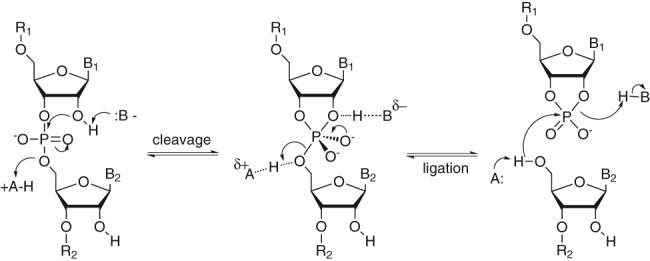
Hairpin ribozyme mechanism (from *left* to *right*) nucleophilic attack followed by intermediate formation and release of newly formed 5′- and 3′-termini (see text for details); (R_1_, R_2_) oligonucleotide chain, (B_1_, B_2_) nucleobase, (A) acid, (B^−^) base.

Hairpin ribozyme catalyzed RNA cleavage and ligation reactions follow a transesterification mechanism (Cochrane and Strobel 2008). Cleavage occurs by nucleophilic attack of the 2′-oxygen on the neighboring phosphorous resulting in a trigonal-bipyramidal intermediate. Upon release of the 5′-OH-group, a 2′,3′-cyclic phosphate is formed. Ligation follows the same reaction path in opposite direction and proceeds via ring opening of the cyclic phosphate, exclusively delivering the natural 3′,5′-phosphodiester (Scheme 1). Ligation is enthalpically favored over cleavage, because ring strain energy is released when opening the cyclic phosphate. Entropically, ligation is disfavored, owing to the decrease in degrees of conformational freedom. However, the entropic cost of ligation is rather small and can be compensated by the favorable enthalpic contribution (Hegg and Fedor 1995, Nahas et al. 2004). In addition, ligation is about two times faster than cleavage (Liu et al. 2007). Thus, the internal equilibrium of the hairpin ribozyme is shifted toward ligation. Translated into practical use this means that the two activities can be controlled by structural modulation. Hairpin ribozymes that form a stable structure, such that fragments remain bound, favor ligation, whereas hairpin ribozymes that are less stable, such that cleavage fragments can easily dissociate, favor cleavage (Fedor 1999, Welz et al. 2003). These characteristic features distinguish the hairpin ribozyme from other small ribozymes, and we have shown in previous work that structural manipulation of hairpin ribozyme variants allows tuning of cleavage and ligation activity (Welz et al. 2003; Ivanov et al. 2005; Vauleon et al. 2005; Drude et al. 2007, 2011; Pieper et al. 2007; Petkovic and Müller 2013, Balke et al. 2014). Among these variants is a hairpin ribozyme that can cleave off its 5′- and 3′-end (Pieper et al. 2007). The cleaved product has two reactive ends that can ligate to circular species or concatemers of two or more molecules.

Herein we address the question whether it is possible to design entirely self-reactive RNAs to efficiently circularize OR polymerize to large RNA entities. In contrast to previous work, our purpose was to optimize RNA sequences for particular conformations favoring monomeric variants or multimerization, rather than tuning the cleavage and ligation rate itself. Self-reactive RNA molecules changing their properties by circularization or increasing their length by polymerization provide a good case-study to exploit the repertoire of state of the art computational design algorithms and to improve them by experimental verification. Good heuristics to embed catalytic activity into RNA molecules with desired functions are highly amendable for synthetic biology, since RNA cleaving or ligating ribozymes constitute an additional layer of gene regulation. Additionally, successful designs would have direct implications on the RNA world theory to explain the emergence of RNA genomes in an early RNA world.

**SCHEME 2.**
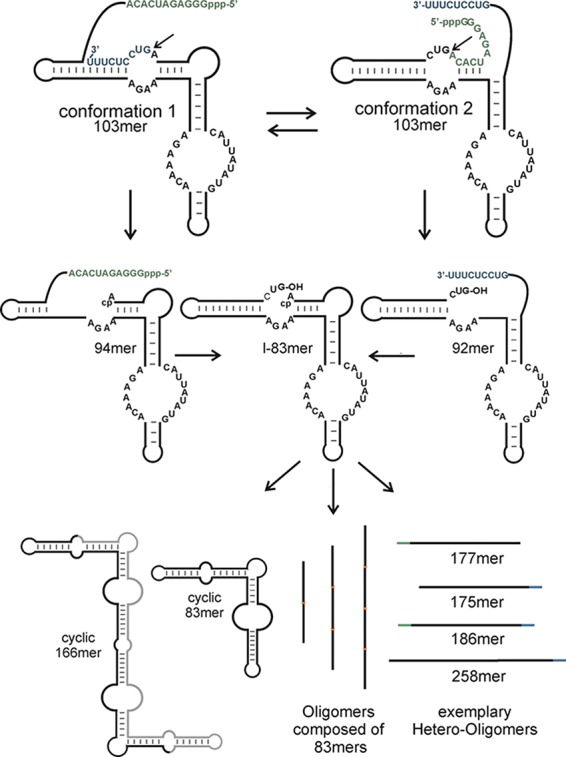
RNA self-processing pathway of CRZ-2. The pathway also applies to designed sequence variants PBD1 to PBD4 (see below). Note that fragment lengths differ for PBD3 and PBD4. RNAs are programmed to fold in two distinct conformations (*top*). Both conformations favor cleavage, such that either the 5′-terminus (green) or the 3′-terminus (blue) can be cleaved off, resulting in a 94- or 92mer (*middle*). These intermediates can refold in the conformation required for cleavage of the remaining 3′- or 5′-end, respectively. The final cleavage product is always an 83mer, which can undergo intramolecular ligation to a circular species (*bottom*, *left*) or self-concatemerize by intermolecular ligation (*bottom*, *middle*). In addition, the fragments resulting from the first cleavage contain either the 2′,3′-cyclic phosphate or the 5′-OH group required for ligation, such that they can also oligomerize with each other or with one or more 83mers (*bottom*, *right*).

Using the ribozyme CRZ-2 (Scheme 2), which was developed previously (Pieper et al. 2007) and recently analyzed in detail (Petkovic and Müller 2013), as template, we computationally optimized sequences using the program switch.pl (Flamm et al. 2001) of the Vienna RNA package (Lorenz et al. 2011). Four variants with different behavior according to our scoring functions were selected and analyzed in detail by polyacrylamide gel electrophoresis (PAGE) and atomic force microscopy (AFM). We present high resolution AFM images of the reaction mixtures, visualizing even the rather short 83mer RNA fragment.

## RESULTS

### Computer-aided sequence design

**FIGURE 1.**
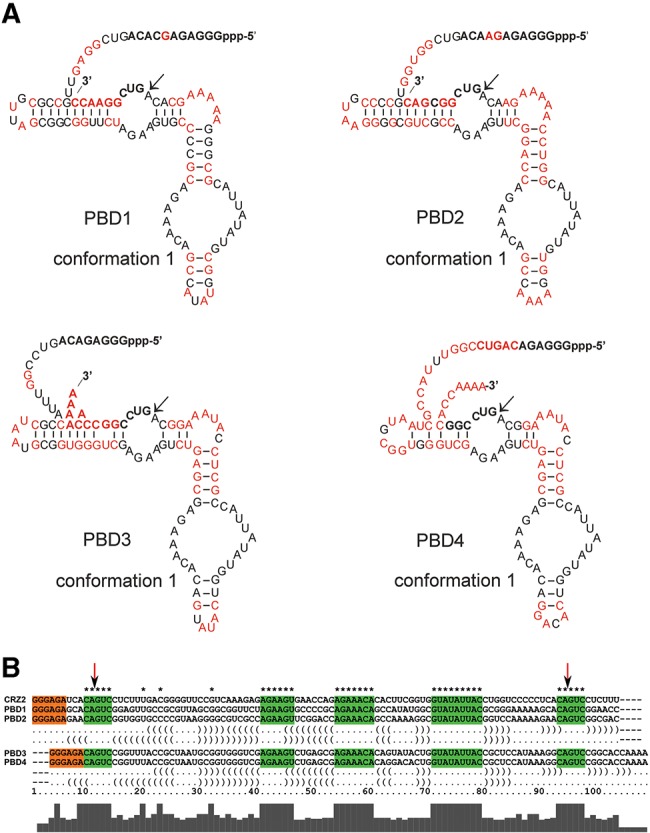
(*A*) Secondary structures of CRZ-2 and PBD 1–4 shown in one of the two possible conformations (cf. Scheme 2). Sequence changes in comparison to the reference RNA CRZ-2 are shown in red. (*B*) Sequence alignment of the four designed RNAs PBD1–4 with the reference system CRZ-2. Green interior loop areas are reported to be essential for cleavage/ligation activity and were therefore fixed during the design process. The orange colored T7 RNA promoter sequence was needed for experimental implementation. The secondary structure in dot-bracket notation *below* shows the constraints on a structural level.

Compared with manual design that we had applied in previous work (Pieper et al. 2007; Petkovic and Müller 2013), computer-aided design is a more sophisticated way toward control of self-processing activity of RNA species. Therefore, we have started a bioinformatics approach to evolve hairpin ribozyme derived RNAs with self-processing activity. We have designed two classes of ribozyme species: Members of the first class should process themselves efficiently into circular monomers, whereas members of the second class would maximize the yield of ligation competent dimers. The design process is complicated by the fact that multiple constraints exist on both sequence and structure level. On the sequence level we included two well-conserved interior loop regions from the hairpin ribozyme (Berzal-Herranz et al. 1993), as well as a T7 RNA promotor sequence at the 5′-end for experimental implementation. On the structural level, the constructs have to be bistable, forming two distinct catalytic centers to cleave off both the 5′- and 3′-ends as depicted in Figure 1 and Supplemental Figure S1. Our approach is a two-step process that first computes a large set of RNA sequences with catalytic properties, and second scores these sequences to select for ribozymes with the desired behavior. Previously, we have shown that the efficient design of bistable molecules is surprisingly easy (Flamm et al. 2001). The algorithm, implemented in the program switch.pl of the Vienna RNA package, mutates initially random sequences into bistable switches *via* consistent mutations guided by a dependency graph. The mutations are meant to increase the probability of forming catalytically active structures and influence the conformations formed upon dimerization of the individual species. Using switch.pl, ∼10,000 bistable RNA molecules conforming to the above design objective were designed and ranked according to two scoring functions κ_1_ and κ_2_ (Equations 2 and 4 in Materials and Methods) that evaluate the probabilities of forming reactive structures and the fraction of circular species in equilibrium. Results of the scoring function for all four sequences and the reference system CRZ-2 can be seen in Table 1. A lower κ_1_-value indicates high catalytic activity of all monomeric variants; a lower κ_2_ indicates a high probability of forming catalytically active homo-dimers. Hence, κ_2_ was used to discriminate between ribozymes that are meant to favor formation of cyclic dimers (lower κ_2_) and those that do not (higher κ_2_). Detailed explanation and formulas can be found in Materials and Methods and Supplemental Figure S2a,b. Figure 1 shows four bistable ribozyme sequences (PBD1 to PBD4) that were selected for experimental validation in comparison to the reference system CRZ-2. Table 1 summarizes their expected properties and the results from the scoring functions (rounded to two decimal figures).

**TABLE 1.**
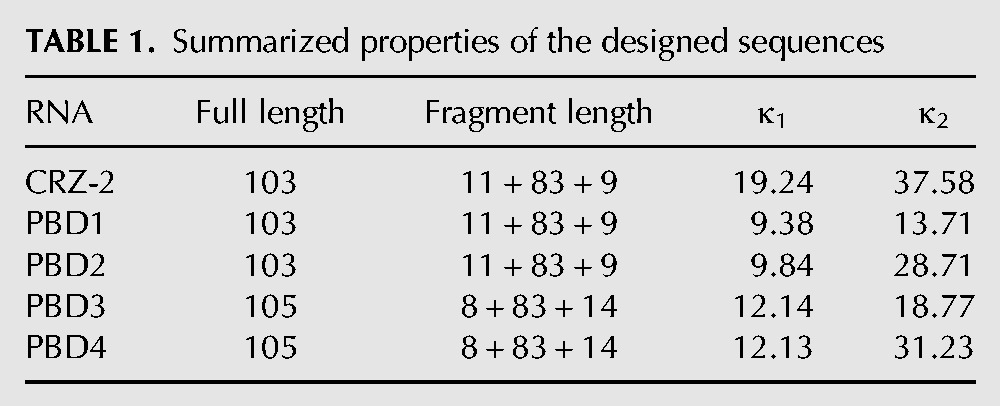
Summarized properties of the designed sequences

Compared with the reference RNA CRZ-2, PBD1–4 differ by various base replacements in the nonconserved regions (Fig. 1). However, the new designed ribozymes were meant to undergo the same cleavage cascade reaction as described for CRZ-2 previously (Petkovic and Müller 2013) and depicted in Scheme 2. Dimerization of the hairpin ribozyme has been demonstrated previously (Butcher and Burke 1994), and is an essential prerequisite for the formation of concatemers by CRZ-2. We therefore assumed that sequences forming catalytically active, intermolecular ligation competent dimers favor concatemerization (PBD1 and PBD3), while sequences that have lower tendency to form these structures, are assumed to predominantly form cyclic monomers (PBD2 and PBD4). Our scoring functions furthermore indicate that PBD1 and PBD2 show increased efficiency to form cyclic monomers (Table 1, κ_1_) compared with PBD3 and PBD4.

### Biochemical analysis of the self-processing behavior of the designed sequences

**FIGURE 2.**
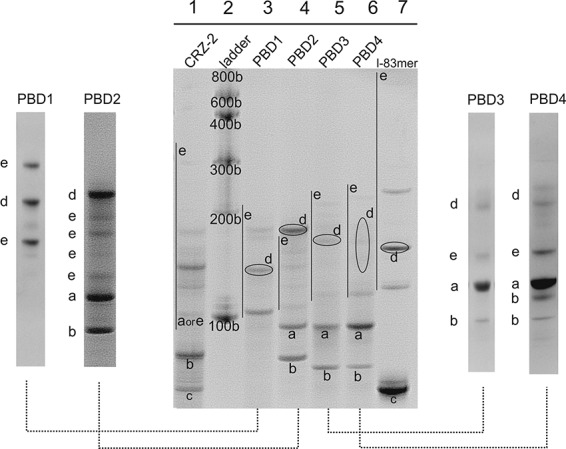
Analysis of self-processing reactions of sequences PBD1-4, CRZ-2, and the linear 83mer (l-83mer) in a 15% denaturing polyacrylamide gel, lane *2*: RNA size standard. For better visualization of individual bands, self-processing reactions of PBD1–PBD4 were analyzed separately with a higher amount of sample loaded onto the gel (separate lanes *left* and *right* to the gel. Note that large scale analysis was carried out separately for each of the designed RNAs PBD1–PBD4. Therefore, the relative positions of bands are not directly comparable between individual gels. Compare also Supplemental Figure S3. Bands were assigned as follows: full-length transcripts (a); cleavage intermediates (5′- or 3′-truncated transcripts) (b); final cleavage product (5′- and 3′-truncated transcripts) (c); cyclic RNA resulting from intramolecular ligation of species c (d); concatemers resulting from intermolecular ligation of species b and c (e).

**FIGURE 3.**
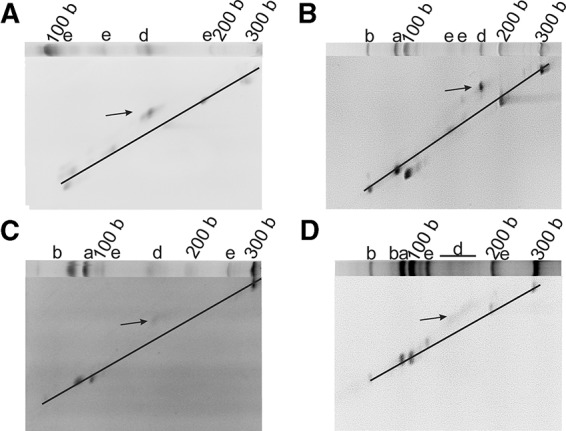
Two-dimensional gel electrophoretic analysis of PBD1 (*A*), PBD2 (*B*), PBD3 (*C*), and PBD4 (*D*). All samples were mixed with a linear RNA size standard prior to subjecting onto the gel. The first dimension gel of the respective system is implemented in each panel. The diagonal marks the linear RNAs; circular species are denoted by an arrow.

**FIGURE 4.**
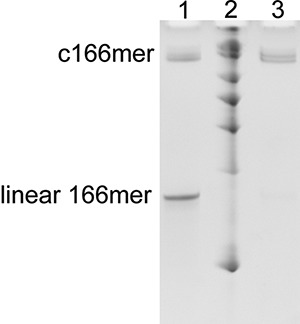
Enzymatic ligation of the inactive 166mer and treatment with exonuclease RNase R. Lane *1*: ligation mixture composed of ligation product and remaining nonligated linear transcript; lane *2*: RNA size standard, 100 bases, 200 bases, 300 bases, 400 bases, 600 bases, 800 bases, and 1000 bases; lane *3*: ligation product mixture after treatment with RNase R (for details see Supplemental Material).

The five RNAs, CRZ-2 and PBD1 to PBD4 were prepared by in vitro transcription with T7 RNA polymerase (see Supplemental Material) and incubated at conditions favoring self-cleavage followed by ligation (Petkovic and Müller 2013). First, reaction products were analyzed using denaturing polyacrylamide gels (Figs. 2, 3, 4). Bands in the gel were visualized by ethidium bromide staining. Table 2 shows the lengths (in number of bases) of products that theoretically can be formed; Figure 2 shows an overview of reactions of all self-processing ribozymes (CRZ-2, PBD1–PBD4). For comparison, the linear 83mer (l-83mer) resulting from two cleavage events in CRZ-2 and being incapable of further cleavage was isolated and incubated at identical conditions (Fig. 2, lane 7). The behavior of this 83mer was analyzed in detail recently (Petkovic and Müller 2013), such that the band pattern produced by the l-83mer could be used as guideline to navigate through the PAA gel and, with the aid of the 2D-gel electrophoresis results (see below), to assign the obtained bands to individual RNA species. This becomes especially important, since chemical modifications at RNA ends (such as OH, phosphate or cyclic phosphate), RNA sequence itself, and RNA structures formed in spite of denaturation can affect the migration behavior of RNA molecules. The standard length marker (lane 2) can only serve as an approximate guideline for higher ligation products.

**TABLE 2.**
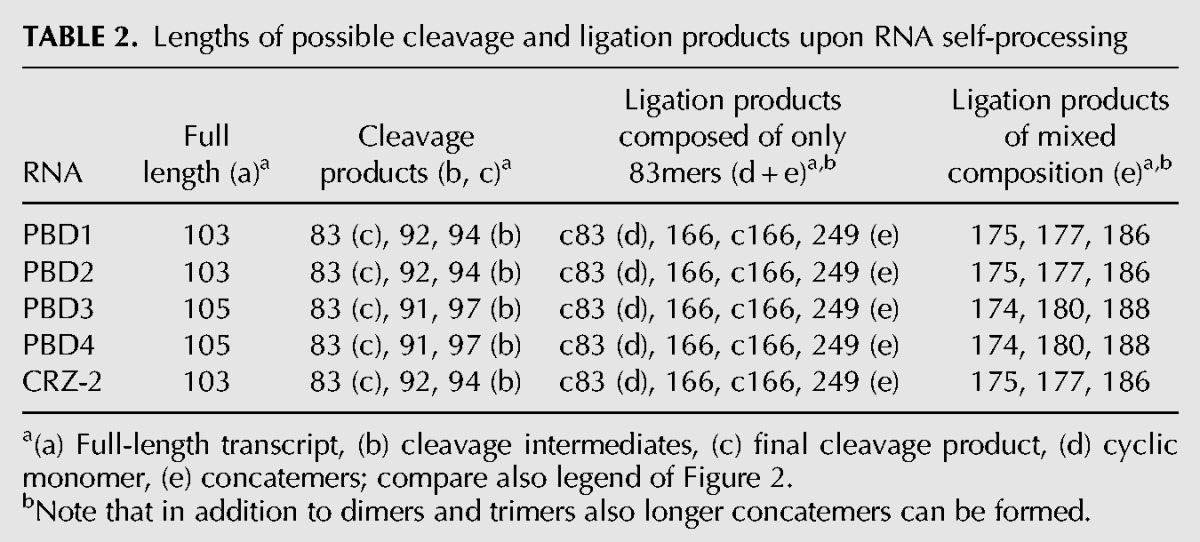
Lengths of possible cleavage and ligation products upon RNA self-processing

#### Full-length transcripts (**a**—103/105mers)

The 103 (CRZ-2, PBD1 and PBD2) or 105mers (PBD3 and PBD4) are typically located below the 100 nt size standard mainly due to the triphosphate at the 5′-end resulting from in vitro transcription of the ribozymes (Fig. 2; Supplemental Fig. S3). The 103mer of CRZ-2 and PBD1 is barely detectable after ribozyme reaction (Fig. 2, lanes 1 and 3), whereas full length transcripts of PBD2 to PBD4 (lanes 4, 5, and 6) are still visible. This implies that CRZ-2 and PBD1 exhibit higher activity in cleaving off the 5′- or the 3′-end or both, and producing the shortened fragments denoted with ***b*** and ***c*** (Table 2; Fig. 2).

#### Cleavage products (b—97, 94, 92, 91mer and c—linear 83mer)

In CRZ-2, PBD1 and PBD2, a 92mer and a 94mer are produced as intermediates upon the first cleavage. These two intermediates occur as one band, since the 94mer carries additional charges from the triphosphate at the 5′-end. For CRZ-2 and PBD2 (Fig. 2, lanes 1, 4), a prominent 92/94mer band is visible, PBD1 shows none of these species. PBD3 and PBD4 produce a 91mer and a 97mer, with the 91mer carrying the triphosphate. Both systems show 91mers, whereas the 97mer is only detectable for PBD4 (lanes 5, 6, and gel pieces shown on the right). The final cleavage product of all test systems is a linear 83mer. Lane 7 shows the 83mer from CRZ-2 used as an additional size standard (Petkovic and Müller 2013). Interestingly, only CRZ-2 shows a band corresponding to the final cleavage product, while linear 83mers of PBD1-PBD4 are not detectable, suggesting an immediate consumption in ligation reactions.

#### Intramolecular monomeric ligation (d—cyclic 83mer)

From all produced monomers (l-83mer, 91mer, 92mer, 94mer, 97mer, 103mer, and 105mers) the linear 83mer is the only RNA that may perform cyclization due to the chemical constitution at its 3′- and 5′-end. However, the migration behavior of an unknown cyclic species in a PAA gel is impossible to predict by common size markers, since the overall shape and the migration behavior of the cyclic RNA strongly depend on the sequence (Grabowski et al. 1984; Sigurdsson and Eckstein 1996). Previously, we have set up a two-dimensional-PAA gel electrophoresis assay (see Experimental section of the Supplemental Material, and Petkovic and Müller 2013) to identify cyclic species by means of their nonlinear movement at different PAA concentrations. While linear species move on a diagonal in the second dimension, cyclic species are expected to show irregular movement. Full-length CRZ-2 appears to form, if at all, only traces of a circular 83mer (Fig. 2, lane 1; cf. also Supplemental Fig. S4), while incubation of the isolated linear 83mer of CRZ-2 alone (lane 7) clearly produces the cyclic species. The cyclic 83mer is located approximately at the 150 nucleotide size standard. For PBD1-PBD4, identification of cyclic species was possible by 2D gel electrophoresis (Fig. 3). Interestingly, all newly designed RNAs (PBD1-PBD4, lanes 3–6) do show circular 83mers, while not showing any linear 83mers. In case of PBD4, the cyclic species is not represented by a discrete band, but rather appears as a smear.

#### Higher noncyclic ligation products (e—dimers, trimers, concatemers)

Intermolecular backbone ligation can only occur upon dimerization of the 83mer and/or the intermediate cleavage products, carrying the required termini (5′-OH and 2′,3′-cyclic phosphate). A summary of these species can be seen in Table 2.

Identification of monomeric cleavage products was straight forward since they move roughly according to their size. Identification of higher ligation products is challenging, because their movement can be irregular (Cruz-Reyes et al. 1998). However, by means of the l-83mer marker (Fig. 2, lane 7) we know that the species moving ∼150 nt length is actually the circular 83mer, while the covalently linked linear 166mer (83 + 83) is located roughly at 120 nt length. Bands above the 200 bases ladder correspond most likely to 249mers (83 + 83 + 83) and even longer molecules. In comparison, we do see multiple species between 100 and 200 nt in the full-length CRZ-2 lane (Fig. 2, lane 1). We can clearly identify the 166mer at the same height as the 166mer in the lane of the l-83mer reference marker (lane 7). Shortly above is a stronger band indicating intermolecular ligation of the 83mer with a 92/94mer, or of the 92mer with the 94mer, respectively. The ratio between linear 166mer and 175/177mer would also be similar to the observed ratio between 83mer (***c*** in lane 1) and 92/94mer (***b*** in lane 1). The bands further up are hard to interpret and might show a little of c83mer and 186mer (92 + 94), as well as a 258/260mer (83 + 83 + 92/94) next to the 300 bases ladder.

Assignment of bands becomes more difficult for PBD1 to PBD4. PBD1 (lane 3), our most efficient ribozyme concerning 5′- and 3′-end cleavage, shows two bands in addition to the c83mer, which most likely correspond to the linear 166mer (83 + 83) and 249mer (83 + 83 + 83), respectively. PBD2 (lane 4) shows four species between the 105mer (***a***) and the c83mer (***d***). Since we can see a clear band for 92/94mers (***b***) we suggest that these species took part in intermolecular ligation reactions with 83mers resulting in a diverse set of dimers (***e***). However, we cannot exclude that a low running trimer is present as well. PBD3 and PBD4 (lane 5, 6) show mostly the same species with different intensities. Analogous to PBD1 they show noncircular species that most likely represent the homo-dimer (83 + 83) and possibly hetero dimers and/or a trimer.

#### Cyclic dimer formation

With the purpose of identifying cyclic dimers in the reaction mixture we designed and synthesized an inactive dimer (CRZ*) which should mimic the behavior of its CRZ-2 equivalent (Supplemental Material). Figure 4 shows two versions of nonreactive CRZ*, the linear species at ∼166 nt length and the enzymatically ligated circular version at a height of ∼800 bases (lanes 1 and 3). By comparison with the results shown in Figure 2, a band at comparable height (∼800 bases), is detected only in the l-83mer marker (lane 7), although being rather weak. All other ribozymes do not exhibit measurable amounts of circular dimers in PAA gels.

### AFM measurements

To obtain deeper insight into the self-processing behavior of the designed RNAs and in particular into chain lengths distribution, we supplemented the biochemical analysis by Atomic Force Microscopy (AFM). AFM imaging is able to visualize RNA chains on single molecule level (Henn et al. 2001), allowing to characterize even rarely produced RNA species that are difficult (if not impossible) to observe in gel electrophoretic experiments. We analyzed four candidates out of the investigated self-processing RNAs with AFM imaging under semidenaturing conditions: CRZ-2, PBD1, PBD4, and the isolated linear 83mer of CRZ-2. These reaction mixtures showed high diversity upon biochemical analysis (Fig. 2, lanes 1, 3, 6, 7), with PBD1 forming predominantly cyclic 83mers, linear 166mers, and 249mers, and PBD4 expressing a plethora of dimeric and of multimeric species.

**FIGURE 5.**
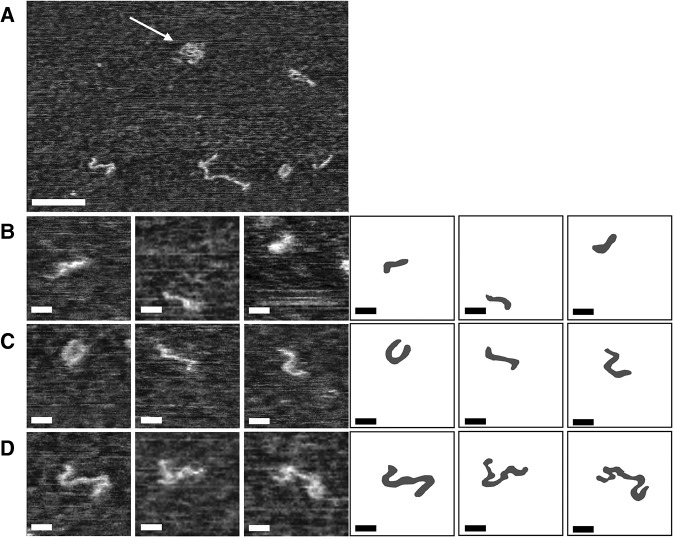
Tapping mode (TM) AFM phase images (range: 0°–30°) of the reaction products resulting from incubation of the l-83mer (isolated from CRZ-2 system) in cleavage/ligation buffer. For AFM analysis, samples were precipitated and resolved in 25 mM EDTA and 3.5 M urea (semidenaturing conditions). Scale bars: 50 nm (*A*), 10 nm (*B*–*D*). The overview scan (*A*) shows RNA chains in coiled (white arrow) and unwrapped conformation. High-resolution TM images (*B*–*D*) allow investigation of the internal structure of 83mers (*B*), dimers (*C*), and trimers (*D*). For convenience, schematics have been included on the *right* side to help with the interpretation of the AFM images. The corresponding height images are given in Supplemental Figure S7.

**FIGURE 6.**
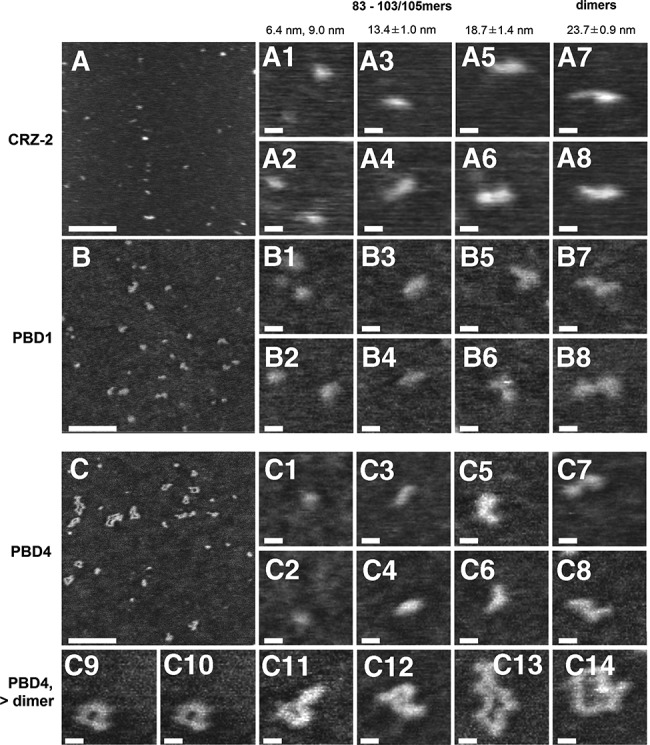
AFM images of RNA: CRZ-2 (*A*, a1–a8), PBD1 (*B*, b1–b8), and PBD4 (*C*, c1–c14). Scale bars: 100 nm (*A*–*C*), 10 nm (a1–c14); height scale 1 nm in all images. RNA chains have typically a height of ∼0.4 nm in the AFM images. Overview scans (*A*–*C*) show a mixture of RNA chains of different contour length *L*_C_ for all three sequences investigated. Analysis of contour-length histograms (see Supplemental Fig. S5 and S6) for CRZ-2, PBD1, and PBD4 allowed association of most of the observed RNA chains with the species listed in Table 2 (as indicated in the figure). This procedure failed for two shortest species (a–c 1,2) and the one having a contour length ∼18.7 nm (a–c 3,4), as the calculated numbers of bases did not match any entry of this table (see text for a detailed discussion).

Figures 5 and 6 show representative examples for tapping mode (TM) AFM images of ribozymes (recorded in air after RNA immobilization on mica and drying). The observed RNA chains adopt either a coiled (see white arrow in Fig. 5A), or uncoiled conformation which consists of rod-like segments, connected by kinks. Hence, for the uncoiled conformation it is possible to measure the lengths of the constituting segments as well as the contour length of the whole chain. That allows a comparison of the observed molecules with secondary structure prediction of the species listed in Table 2. Immobilized under native conditions, all observed molecules had a coiled conformation (data not shown), while semidenaturing conditions resulted mostly in uncoiled conformations having the rod-kink-motif. Hence, the majority of the AFM measurements were done on RNA chains prepared under semidenaturing conditions (see Figs. 5, 6 for a representative overview). Histograms showing both the contour lengths and the segment lengths for all four analyzed ribozymes can be seen in Supplemental Figures S5 and S6, contour-length results are summarized in Table 3. These histograms are in agreement with the expected values from secondary and tertiary structure prediction: All single-molecule ribozyme species (83mer–105mer) are expected to form a reactive structure with two stiff helical regions (segments) connected with a flexible kink. If we assume a typical pitch of 0.3 nm per base pair (Arnott et al. 1973; Henn et al. 2001), the 83mer consists of two stiff regions with 5.4 and 6.3 nm length plus a kink of about five bases. The contour length would therefore be ∼11.7 nm plus the kink region. Monomers that have noncleaved ends would form the same helices but have additional single stranded regions in the kink region or sticking out from one of the helices. Based on these single stranded regions, different monomer variants would be hardly distinguishable with AFM imaging. Accordingly, different dimer species are expected to fold into a conformation where ∼166 bases are involved in successive helical regions (166 × 0.15 nm = 24.9 nm), trimers with 249 bases resulting in 37.35 nm and so on.

**TABLE 3.**
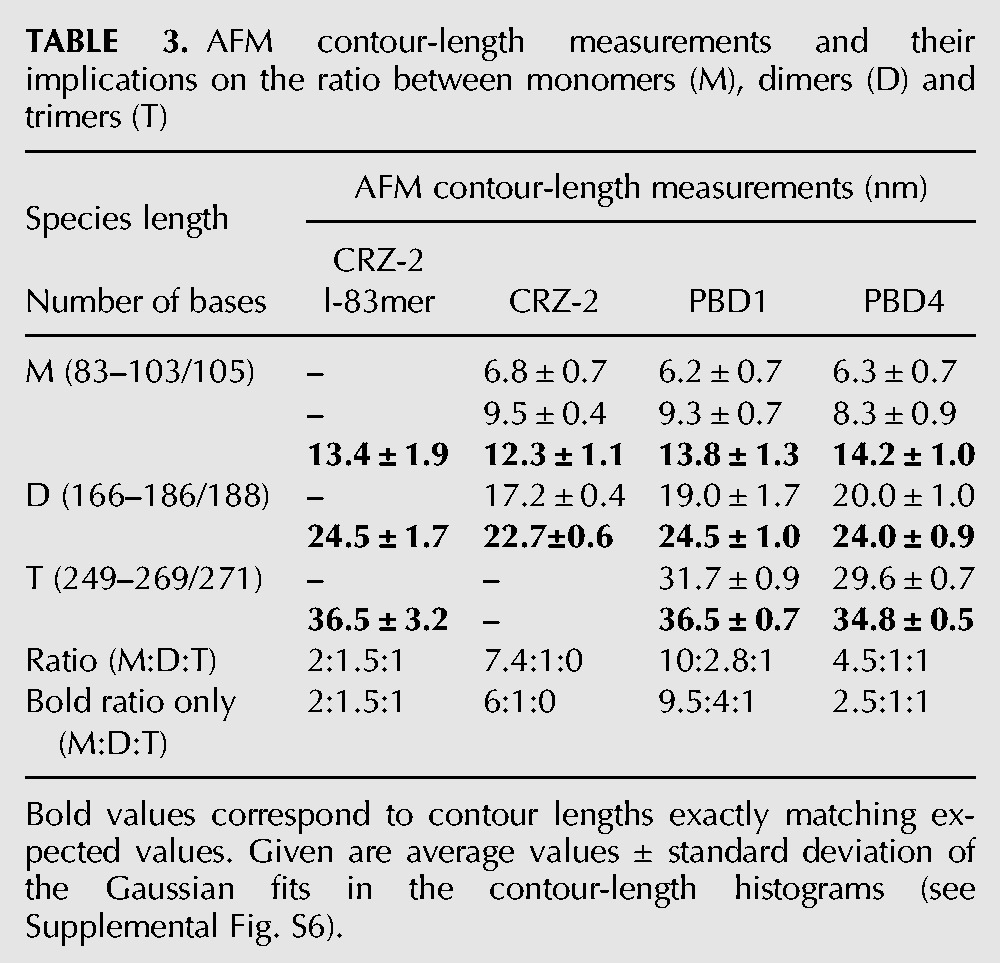
AFM contour-length measurements and their implications on the ratio between monomers (M), dimers (D) and trimers (T)

The contour-length histogram of the linear 83mer shows three contour-length peaks at 13.4, 24.5, and 36.5 nm, as well as very few molecules with even higher lengths (Supplemental Fig. S6a). These peaks closely map to expected values for regularly folded monomers, dimers and trimers, respectively, making an interpretation straightforward. The segment lengths, representing individual helical regions, showed peaks at 5.9, 8.5, and 13.8 nm (Supplemental Fig. S5a), which most likely correspond to the monomer helices and dimer-variants of these helices. AFM imaging reveals that the monomer of the l-83mer typically consists of one “short” and one “long” segment, which enclose an angle of ∼110° (Fig. 5B). The dimer may be composed of two, three or even four segments (Fig. 5C, left to right), while the trimer shows typically a very complicated internal structure (Fig. 5D). Hence, a large variety of possible conformations is observed for dimers and trimers in the AFM images.

Full-length CRZ-2, as well as PBD1 and PBD4 show an even wider spectrum of contour-length peaks and chain conformations (see Supplemental Figs. S5, S6), which is expected from the computational design and the biochemical analysis. In the AFM measurements, full-length CRZ-2 creates predominantly species being shorter than 24 nm (Supplemental Fig. S6b). This “cut-off” shifts to 36 nm for PBD1, while much longer RNA chains (up to ∼80 nm) are observed for PBD4. Hence, the population shifts progressively to longer RNA products from CRZ-2 over PBD1 to PBD4, which is in agreement with the gel electrophoretic analysis. All structures show contour-length peaks at expected values close to those from the l-83mer, which makes an identification of monomers, dimers, and trimers straightforward. Monomers appear mostly in a rod-like conformation, and a kink (similar to the l-83mer monomers) is rarely resolvable (Fig. 6, a3, a4, b3, b4, c3, c4). Dimers adopt L- and Z-like conformations (Fig. 6, a7, a8, b7, b8, c7, c8). Higher ligation products (trimers, etc.) are currently only observed for PBD4, which can lead (similar to the l-83mer) to very complicated and irregularly shaped internal chain structures (c9–c14 in Fig. 6). However, besides these species, also additional peaks are found at contour lengths that are (i) shorter than the expected value for a regularly folded monomer (6.5 and 9.0 nm, observed for CRZ-2, PBD1 and PBD4), (ii) between the monomer and dimer length (17.2 nm for CRZ-2, 19.0 nm for PBD1, and 20.0 nm for PBD4), or (iii) between the dimer and trimer length (31.7 nm for PBD1, 29.6 nm for PBD4). We can exclude that cleaved ends from processed full-length ribozymes would have a length of 6.8 nm or 9.5 nm in the AFM images (for the measurement conditions used in the experiments). Instead, we can show that the smaller peaks match very well to segment length measurements (Supplemental Fig. S5), suggesting that only one of the two helices is resolved by AFM imaging. Accordingly, we know that the catalytically active structure involves tertiary interactions to closely orient both helices to each other. Uncleaved structures with single stranded regions in the kink region might favor the back folding of the helices despite semidenaturing conditions, which are meant to destroy tertiary base pairs. The AFM images further support this interpretation: Species having a contour length ∼18.7 ± 1.4 nm (i.e., between the monomer and dimer length) typically show an L-like conformation. Complementing this chain structure with a third segment (which might be irresolvable in the images due to back folding of one helix) having a length of 6.6 ± 0.4 nm (first peak in the segment length histograms, see Supplemental Fig. S6) gives a *Z*-like conformation with a total contour length of 25.3 ± 1.8 nm, matching well the expected value for a regularly folded dimer (24.9 nm). Using the same reasoning, the peaks ∼6.4 ± 0.3 and 9.0 ± 0.6 nm may be interpreted as “partially back folded” monomer and the one ∼30.7 ± 1.5 nm as a “partially back folded” trimer.

To compare the AFM findings with the computational design and the results of the biochemical analysis, we calculated the number frequency of each species from the contour-length histograms. The ratio between observed monomers, dimers, and trimers are given in the last two lines of Table 3: One line calculates the ratios regarding all peaks and one line regards only those peaks in the contour-length histograms that exactly matched the expected contour lengths. However, both lines show very similar ratios, indicating that using the “backfolding hypothesis” does not affect the final conclusions of the AFM measurements.

## DISCUSSION

Taken together, the results of the biochemical analysis in combination with AFM imaging confirm the predicted behavior of the self-processing RNAs CRZ-2, PBD1, PBD2, PBD3, and PBD4. All RNAs undergo two initial cleavage events that truncate the full length transcript at the 5′- and 3′-end to a linear 83mer with 5′-hydroxyl group and 2′,3′-cyclic phosphate required for ligation. The subsequent intramolecular ligation delivers exclusively cyclic versions of the 83mer, whereas intermolecular ligation produces dimers and longer concatemers, which apart from PBD4, have no or rather low tendency toward cyclization. This implies that formation of cyclic dimers is extremely unfavored, since it requires the ligation at two sites simultaneously. The same applies to longer concatemers that are rather rare anyway.

**FIGURE 7.**
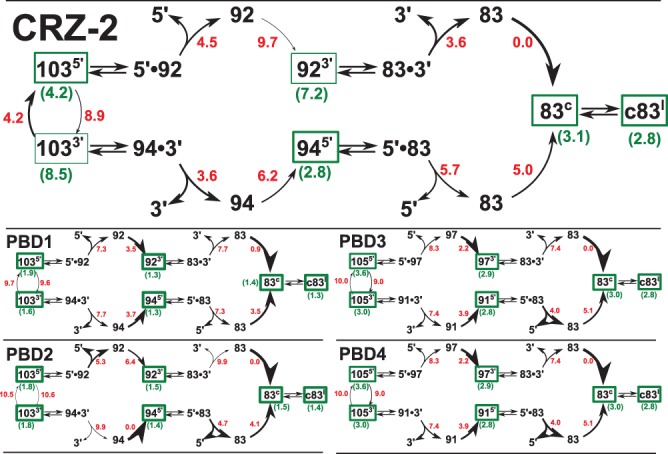
Cleavage cascades of molecules CRZ-2 and PBD1-4. Black numbers correspond to the length of the molecules or to the fragment to be cleaved (5′- and 3′-end). Superscripts 5′, 3′, c, or l mark molecules in a reactive conformation to cleave the 5′-end, the 3′-end, to circularize, or to linearize, respectively. Reversible cleavage reactions are indicated by double arrows, bent arcs denote refolding steps that are considered as irreversible. The line width of arcs is proportional to the refolding rate; red numbers denote the corresponding energy barriers (limiting the refolding rate). The line width of green boxes is proportional to the equilibrium probability of a reactive conformation; green numbers in parentheses denote the corresponding difference between the free energy of the reactive structure and the ensemble free energy.

Comparing experimental results with theoretical predictions, we observed two major points, as discussed in detail below. (i) All designed species are highly efficient in circularization or ligation of cleavage products (optimized with the scoring function κ_1_). Thermodynamic optimization, however, resulted in less efficient cleavage reactions compared to CRZ-2, since the cleaved ends remain tightly bound and equilibrium is shifted toward ligation. (ii) PBD1–PBD4 vary in the formation of monomers and multimers (as intended by scoring function κ_2_), but interestingly, molecule optimization for stable dimers reduced the variety of dimer species and did not lead to a higher yield of trimers. Figure 7 shows a detailed analysis for each ribozyme and will serve as a guideline to discuss observed results from PAA gel electrophoresis and AFM. During the cleavage cascade, we can distinguish three types of reaction steps: (i) formation of reactive structures, (ii) dissociation of cleaved ends after ribozyme reaction, and (iii) refolding of an unbound reaction product into a new reactive structure. In Figure 7, each of these steps is characterized by an activation free energy (see Supplemental Material for details).

### Full-length transcripts (*a*—103/105mers)

Our theoretical analysis shows that dissociation of the cleaved ends from computationally optimized ribozymes (PBD1–4) has to overcome a higher energy barrier than in the case of manually designed CRZ-2 (Fig. 7). This is due to the fact that designed molecules are optimized to fold primarily into catalytically active conformations, and therefore also the cleaved conformations with tightly bound ends are very stable. It is known that tightly bound fragments shift equilibrium toward ligation (Fedor 1999), and this would explain why we see full-length product for three of our four designed ribozyme species (PBD2, PBD3, and PBD4), but not for CRZ-2. Here, the 5′-end is efficiently cleaved off, and the resulting transient fragments tend to accumulate as 92mers (Fig. 2, lane 1). The full-length transcript PBD1 (103mer) is completely consumed despite its high dissociation barriers, indicating that in agreement with theoretical analysis, the dissociation of cleaved ends is an irreversible step at experimental conditions.

### Cleavage products (*b*—97, 94, 92, 91mer, and *c*—linear 83mer)

The cleavage cascade can start with either of two reactive conformations resulting in cleavage of the 5′- or 3′-end. In the case of CRZ-2, none of these conformations correspond to the ground state of the molecule, rather they are 4.2 and 8.5 kcal/mol above the ensemble free energy. Cleavage of the 5′-end is the favored reaction, but results in a structure that has to overcome a high barrier to fold into the reactive 92mer conformation for 3′-end cleavage. In equilibrium, the reactive structure is sparsely populated, since it is 7.2 kcal/mol above the ensemble free energy. We therefore expect that the prominent band ***b*** in the CRZ-2 lane in Figure 2 is mostly 92mer, since the 94mer is (i) the less favored cleavage product and (ii) more likely to undergo the following-up cleavage reaction. PBD3 and PBD4 enable a clear separation of cleavage products on the gel picture, due to their differently sized ends. Both molecules differ by only two point mutations in one hairpin loop of the reactive conformations (Fig. 1). Since this hairpin remains closed in all reactive species as well as on the most favorable refolding paths between the species, all important free energies differ by a constant factor (1.4 kcal/mol), and the barrier heights and structure ensemble probabilities are the same. The distribution of monomeric species should therefore be exactly the same for PBD3 and PBD4. Both molecules favor to cleave first the 3′-end, and second the 5′-end. This is in accordance with experimental results, showing mainly the 91mer as favored intermediate product. The 97mer is observed, particularly for PBD4, when higher amounts of RNA are subjected onto the gel (Fig. 2; Supplemental Fig. S3d).

### Higher noncyclic ligation products (*e*—dimers, trimers, concatemers)

All hairpin-ribozyme variants can form two long helices, both of which have the possibility to form stable dimers that preserve the feature of catalytic activity. PBD1 and PBD3 have self-complementary hairpin loops, which results in a generally stronger tendency to dimerize, and a higher probability to retain catalytic activity upon dimerization. Since PBD1 is extremely efficient during the monomeric cleavage cascade, it shows only minor amounts of intermediate cleavage products (Fig. 2; Supplemental Fig. S3a) that could form dimers. Accordingly, the only dimer species we see is the 166mer (***e*** in lane 3). CRZ-2 and PBD2 show the greatest variety of concatemeric species. In the case of CRZ-2, we see stable 92-/94- and 83mer cleavage products (Fig. 2, bands ***b*** and ***c*** in lane 1). The probabilities to ligate their reactive ends are higher than the probabilities to cleave off the remaining terminal sequence patches, which corresponds to the fact that we observe a variety of concatemeric species. PBD2 shows only the 92-/94mer band ***b*** and no band for the 83mer ***c*** (Fig. 2, lane 4), but it has the highest probabilities (after PBD1) to ligate intermediate cleavage products, and, in contrast to PBD1, a low probability to cleave reactive ends upon dimerization.

PBD3 and PBD4 differ only in the self-complementarity of one hairpin loop and thus should be the best systems to study the influence of such mutations. Resulting from perfect self-complementarity, PBD3 has both higher probabilities to ligate intermediate products and higher probabilities to cleave ends from dimer species. However, the only detectable cleavage intermediate on gel pictures for PBD3 is the 91mer, which cannot ligate with itself to a higher species. Accordingly, we see exclusively the linear 166mer (dimer). For PBD4 also the 97mer is detectable (Fig. 2; Supplemental Fig. S3d). Interestingly, PBD4 shows more multimeric species, suggesting that design toward stable dimers (PBD3) leads to a lower diversity of multimers.

### AFM visualization of RNA molecules

RNA species are identified using differences in their contour length, which however, can cause ambiguities if species differ only by a few nanometers. Hence, it was not possible to distinguish linear and cyclic species (same contour length), different monomeric cleavage products from the full length transcript (contour lengths differ by <2 nm), or to distinguish, which type of dimer, trimer, etc. is observed in the AFM image. However, AFM resolved structural features (helices, loop regions) and observed segment and contour lengths that match with secondary structure prediction for monomers, dimers, and trimers. In the case of the linear 83mer of CRZ-2 (which can only form multiples of itself) the typical pitch of 0.30 ± 0.02 nm per base pair in a helix (Arnott et al. 1973; Henn et al. 2001) matches exactly our observed segment and contour lengths. Contour lengths that do not match the values of regularly folded monomers, dimers, and trimers can be explained by spatial proximity of two adjacent segments, such that two segments appear as one. Supporting this hypothesis, adding a single segment from the segment length histogram to truncated RNA species would lead to expected contour lengths.

We furthermore observed that, although the samples were dried before imaging, the RNA chains kept most of the initial helical conformation. This was also observed in earlier studies (Bonin et al. 2000; Henn et al. 2001; Abels et al. 2005), and indicates that the RNA chain structure is sufficiently conserved to yield meaningful results using AFM imaging in air. Tip convolution, which may lead to a systematic overestimation of the contour lengths, introduces only minor disturbances. Using typical experimental parameters (tip radius <5 nm, RNA chain height <0.4 nm), tip convolution increases the lateral chain extension by <3 nm (measured as full-width half maximum/FWHM) (Ortinau et al. 2010), which is <20% of the monomeric contour length, <10% of the dimeric one, etc. However, the good quantitative agreement suggests that tip convolution effects (i.e., the effective tip radius) are smaller than expected for tip radii extracted from calibration measurements as described in Materials and Methods. Taken together, the results of AFM measurements confirmed and complemented the conclusions drawn from the gel electrophoretic analysis. While smaller fragments are dominating for CRZ-2, a tendency toward larger constructs is seen for PBD1, and for PBD4 a majority of rather complex structures is detected (Fig. 6). Comparing the outcome of the AFM analysis for PBD4 with the gel shown in Figure 2, these complex structures are either cyclic species of varying lengths or folded concatemers, since they most likely correspond to the smear of bands in the area of ***d*** (Fig. 2, lane 4, cf. also Fig. 3D; Supplemental Fig. S4d), and thus show an anomalous migration behavior as typically observed for cyclic or folded RNA species (Grabowski et al. 1984; Sigurdsson and Eckstein 1996).

We obtain clear results from contour-length measurements counting *relative* amounts of monomers, dimers, and trimers (Table 3). Regardless of whether we compare ratios of theoretically expected peaks only, or include the peaks corresponding to partly unresolved molecules, we observe more dimeric and trimeric species for our newly designed species PBD1 and PBD4, which is in agreement with our design objective. Furthermore, PBD1 tends to form dimer species, again in agreement with our design goal, while PBD4, which was theoretically optimized to form cyclic monomers, shows the highest multimer variety both on PAA gel electrophoresis and AFM imaging.

To summarize, imaging ribozymes on the single-molecule level using AFM provides information that complements results obtained from the gel electrophoretic analysis and the computation analysis (and vice versa), making a combination of these techniques promising and very powerful. Our study revealed that (i) self-processing activity can be programmed into RNA structures, (ii) self-processing activity can be predicted and optimized by computer-aided design, and (iii) AFM turned out to be a powerful technique to image the reaction products at the single molecule level, even for short RNAs (<100mer). Dynamic processes like self-induced topology changes and oligomerization and their sensitivity upon sequence variation are essential for biological self-organization and evolution. Moreover, a large number of publications over the past two years have shown that biological processing of RNA into circular species with often still unknown function is widespread in nature. Thus, nowadays the emergence of circular RNAs and their cellular functionalities are actively investigated (Hansen et al. 2011; Abe et al. 2012; Danan et al. 2012; Jeck et al. 2013; Liu et al. 2013), making the development of in vitro techniques for RNA circularization and the study of models mimicking the processing into circular species even more important.

## MATERIALS AND METHODS

### Computational ribozyme design

To have a consistent, length-independent annotation for all possible RNA species that can emerge from a starting (full-length) ribozyme, we introduce the following notation: We denote the 5′- and 3′-ends of the full-length molecule as *L* (left) and *R* (right), respectively, and the linear core as *C* (center). An initial ribozyme species therefore consists of three parts and can be abbreviated as “*LCR*” molecule. Additionally we introduce the term *O* for the circular version of *C*. Despite the ability of *C* to form a circular *O*, multiple copies of *C* can ligate to one long strand that itself can form a maxi-cycle (e.g., *CCC*↔*C*_3_↔*O*_3_).

The following two scoring functions (κ_1_ and κ_2_) were used to select for ribozymes which are able to process themselves efficiently into cyclic monomers (κ_1_) and to differentiate between those, which predominantly form catalytically active or inactive dimers (κ_2_). Both functions estimate rates for cleavage reactions by computing the probabilities of catalytic secondary structures, hence following two hypotheses: First, a cleavage/ligation rate is proportional to the equilibrium probability of a catalytically active secondary structure; second, the cleavage reaction leads to dissociation of the shorter fragment and is therefore irreversible. Equilibrium probabilities of RNA molecules can be calculated from the equilibrium partition function (*Z*); *Z* can be calculated using the McCaskill algorithm (McCaskill 1990) implemented in RNAfold of the Vienna RNA package (Lorenz et al. 2011). Let *Z*(*LCR*) be the equilibrium partition function over all feasible secondary structures compatible with the molecule *LCR,* and *Z*(*LCR*^*L*^) be the constraint partition function over all reactive secondary structures in which *L* can be cleaved off, then the probability *P*(*LCR*^*L*^) can be computed as

(1) $$P(LCR^{L})=\frac{Z(LCR^{L})}{Z(LCR)}$$

All computations were done using the Vienna RNA package Version 2.1.6 with standard energy parameters at 37°C. Supplemental Figure S2a shows our model of the cleavage cascade yielding cyclic monomers. It starts with a full-length molecule (*LCR*) that can process itself into the linear catalytic core in two parallel ways. Either the 5′-end (*L*) of the sequence is cleaved first and the resulting truncated version (*CR*) cleaves the 3′-end (*R*), or vice versa. For both of these parallel, two-step reaction pathways we are interested in the rate limiting step which determines the speed of the cascade. Since we approximate cleavage rates from probabilities of catalytic secondary structures, the rate limiting cleavage reaction is the minimum of both probabilities, and the total rate is the sum of both parallel cleavage pathways. The yield of circular reaction products is computed as the fraction of circular molecules in equilibrium (*Z*(*O*)/(*Z*(*O*) + *Z*(*C*))) resulting in the following scoring function:

(2) $$\kappa_{1}=-\ln\left(\left(\min\left\{ \begin{array}{c} P(LCR^{L})\\ P(CR^{R}) \end{array}\right.+\min\left\{ \begin{array}{c} P(LCR^{R})\\ P(LC^{L}) \end{array}\right.\right)\cdot \frac{Z(O)}{Z(O)+Z(C)}\right)$$

Our model of the cleavage/ligation cascade which forms circular dimers is shown in Supplemental Figure S2b. It follows the assumption that dimerization between full-length molecules happens first, then an intramolecular cleavage cascade is triggered, and finally the system equilibrates between all dimeric cleavage products. While monomers have one reactive ground state with two conserved interior loops to cleave one of their ends, dimers can form up to two reactive centers in three different ways to cleave one end (see Supplemental Fig. S2b). The two interior loops needed for a reaction are commonly called loop A (harboring the reactive site) and loop B. Our computations to score the dimer-cleavage cascade require at least one of these loop regions to be formed intermolecularly, since κ_1_ already scores the molecules according to their intramolecular cleavage efficiency. The probability to cleave both 5′-ends (*L*) from a *LCR* dimer $P(LCR_{d}^{2L})$ can therefore be computed as

(3) $$P(LCR_{d}^{2L})=\frac{Z(LCR_{d}^{2L})}{Z(LCR_{d})},$$

where $Z(LCR_{d}^{2L})$ is the sum of two distinct sets of structures in which loop B is either formed intramolecularly or intermolecularly.

Similar to κ_1_, the yield of circular dimers is computed as the fraction of circular dimers in equilibrium (*Z*(*O*_*2*_)/(*Z*(*CC*) + *Z*(*C*_2_) + *Z*(*O*_2_))) with *CC* and *C*_2_ denoting noncovalently and covalently bound dimers, respectively. The second scoring function κ_2_ is therefore computed as

(4) $$\begin{array}{rl} \kappa_{2} & =-\ln(\frac{{\left[LCR_{d}\right]}_{\theta }}{{\left[LCR\right]}_{\theta }}\left(\min\{\begin{array}{c} P\left(LCR_{d}^{2L}\right)\\ P\left(CR_{d}^{2R}\right) \end{array}+\min\{\begin{array}{c} P\left(LCR_{d}^{2R}\right)\\ P\left(LC_{d}^{2L}\right) \end{array}\right)\cdot \\ & \;\frac{Z\left(O_{2}\right)}{Z\left(CC\right)+Z\left(C_{2}\right)+Z\left(O_{2}\right)}), \end{array}$$

where the first term [*LCR*_*d*_]_θ_/[*LCR*]_θ_ computes the equilibrium ratio between dimers and monomers at a given concentration θ (here 100 nM) for the *LCR* molecule following Bernhart et al. (2006) The scoring function only maximizes the probabilities for catalytically active homo-dimers; pathways that involve dehybridization of partially cleaved species are not included. This corresponds to the assumption that intramolecular cleavage reactions as well as intramolecular folding kinetics are faster than intermolecular structural rearrangements.

### Self-processing reactions

RNAs (11.25 pmol) were solved in Tris–HCl buffer (10 mM, pH = 7.5). After denaturation for one minute at 90°C, RNA folding was allowed for 10 min at room temperature. To initiate the cleavage reaction, MgCl_2_ hexahydrate to a final concentration of 10 mM was added and reaction was allowed to proceed for 2 h at 37°C. To favor ligation, Mg^2+^ concentration was increased up to 50 mM, and reaction proceeded for additional 2 h at 37°C. Reaction was stopped using an equal volume of stop mix composed of urea (7 M) and EDTA (50 mM) for the following PAGE analysis.

### Two-dimensional electrophoresis

Identification of circular RNAs by 2D electrophoresis is based on the fact that the migration of linear and circular nucleic acids is distinctly dependent on the gel pore size (Sigurdsson and Eckstein 1996; Umekage and Kikuchi 2009). To enrich the samples with linear RNAs for better identification of the circular species, a commercially available RNA size standard (RiboRuler low-range RNA ladder; *Fermentas*) being composed exclusively of linear RNAs was added. Each individual mixture was separated in the first dimension gel. Then, the gel piece corresponding to the entire lane was cut out and used for electrophoresis in the second dimension, upon which linear RNAs are supposed to form a diagonal. Covalently closed cyclic RNAs possess reduced degrees of freedom, thus migrating in nonlinear dependence on the linear species and occurring beyond the diagonal (Pasman et al. 1996).

All ribozyme variants (11.25 pmol) were analyzed using 2D PAGE (for polyacrylamide gel composition, buffers and staining see “PAGE analysis”). First dimension: denaturing conditions (7 M urea) 15% polyacrylamide; second dimension: 17.5% denaturing polyacrylamide or 15% native polyacrylamide.

### RNA preparation for AFM analysis

Ribozyme reactions were carried out as described above using 400 nM RNA. After reaction, the product mixture was diluted 1:10, and 5 µL of this solution were lyophilized. The pellet was taken up in 50 µL of semidenaturing buffer (25 mM EDTA, 3.5 M urea) to a final RNA concentration of 4 nM for imaging. Resolved RNA samples were frozen in liquid nitrogen until use.

### Atomic force microscopy (AFM)

AFM imaging was performed in air using a Multimode atomic force microscope (Veeco/Digital Instruments) equipped with a Nanoscope IIIa controller. The AFM piezo scanner was calibrated using calibration gratings TGZ01 (rectangular 26 nm SiO_2_ steps on silicon wafer; MicroMasch) and PG (chessboard-like pattern on silicon, 100 nm depth and 1 µm pitch; manufacturer: Digital Instruments).

RNA samples were prepared by placing a small droplet of RNA solution onto freshly cleaved mica (SPI Supplies). For the investigated RNA constructs, adsorption times of 30 sec to 2 min were sufficient to obtain a suitable RNA surface coverage on the mica substrate. After adsorption, the RNA samples were rinsed in Milli-Q water (Millipore) and dried in a laminar flow hood, followed by AFM imaging.

The images were recorded with conventional Tapping Mode in air using standard tapping mode cantilevers (OMCL-AC160TS, Olympus). Before usage the cantilevers were tested with a Nioprobe self-imaging sample (Aurora Nanodevices) and only cantilevers with tip radius <5 nm were used for imaging. To reduce tip contamination by RNA uptake during imaging process, cantilevers were functionalized with 3-aminopropyldimethyl-ethoxysilane (APDES) from *ABCR* (Karlsruhe) one day prior usage.

In contrast to DNA samples, whose structure often remains unchanged even after storage periods of several months (as judged by their spatial properties in AFM imaging), samples had to be imaged within few days after preparation. The highest resolutions were always obtained directly after preparation, while storing in air often led to post-preparational RNA chain degradation already after few weeks.

Images were analyzed using the software supplemented with the AFM. The shape of an RNA chain was “retraced” in terms of a sequence of connected straight segments, which allowed to calculate the contour length as sum of Euclidean distances (Rivetti et al. 1996). As shown by Rivetti and Codeluppi (2001) (who numerically assessed the accuracy of different methods for contour-length determination) this approach has an intrinsic error <1%. The main source of error in the contour-length determination is therefore given by the lateral resolution of the AFM, which is on the order of few nanometers (see Discussion for details).

## SUPPLEMENTAL MATERIAL

Supplemental material is available for this article.

## Supplementary Material

Supplemental Material

## References

[PETKOVICRNA047670C1] Abe N, Abe H, Ito Y. 2012 Synthesis of dumbbell-shaped cyclic RNAs for RNA interference. Curr Protoc Nucleic Acid Chem Chapter 16: Unit 16.4. 1–11. 10.1002/0471142700.nc1604s48 22395966

[PETKOVICRNA047670C2] Abels JA, Moreno-Herrero F, van der Heijden T, Dekker C, Dekker NH. 2005 Single-molecule measurements of the persistence length of double-stranded RNA. Biophys J 88: 2737–2744. 15653727 10.1529/biophysj.104.052811 PMC1305369

[PETKOVICRNA047670C3] Arnott S, Hukins DWL, Dover SD, Fuller W, Hodgson AR. 1973 Structures of synthetic polynucleotides in A-RNA and A′-RNA conformations: X-ray-diffraction analyses of molecular conformations of polyadenylic acid–polyuridylic acid and polyinosinic acid–polycytidylic acid. J Mol Biol 81: 107–122. 4777303 10.1016/0022-2836(73)90183-6

[PETKOVICRNA047670C4] Balke D, Zieten I, Strahl A, Müller O, Müller S. 2014 Design and characterization of a twin ribozyme for potential repair of a deletion mutation within the oncogenic *CTNNB1*-ΔS45-mRNA. ChemMedChem 9: 2128–2137. 25112518 10.1002/cmdc.201402166

[PETKOVICRNA047670C5] Bernhart SH, Tafer H, Mückstein U, Flamm C, Stadler PF, Hofacker IL. 2006 Partition function and base pairing probabilities of RNA heterodimers. Algorithms Mol Biol 1: 3. 16722605 10.1186/1748-7188-1-3 PMC1459172

[PETKOVICRNA047670C6] Berzal-Herranz A, Joseph S, Chowrira BM, Butcher SE, Burke JM. 1993 Essential nucleotide sequences and secondary structure elements of the hairpin ribozyme. EMBO J 12: 2567–2573. 8508779 10.1002/j.1460-2075.1993.tb05912.x PMC413496

[PETKOVICRNA047670C7] Bonin M, Oberstrass J, Lukacs N, Ewert K, Oesterschulze E, Kassing R, Nellen W. 2000 Determination of preferential binding sites for anti-dsRNA antibodies on double-stranded RNA by scanning force microscopy. RNA 6: 563–570. 10786847 10.1017/s1355838200992318 PMC1369937

[PETKOVICRNA047670C8] Butcher SE, Burke JM. 1994 A photo-cross-linkable tertiary structure motif found in functionally distinct RNA molecules is essential for catalytic function of the hairpin ribozyme. Biochemistry 33: 992–999. 8305446 10.1021/bi00170a018

[PETKOVICRNA047670C9] Cochrane JC, Strobel SA. 2008 Catalytic strategies of self-cleaving ribozymes. Acc Chem Res 41: 1027–1035. 18652494 10.1021/ar800050c

[PETKOVICRNA047670C10] Cruz-Reyes J, Piller KJ, Rusché LN, Mukherjee M, Sollner-Webb B. 1998 Unexpected electrophoretic migration of RNA with different 3′ termini causes a RNA sizing ambiguity that can be resolved using nuclease P1-generated sequencing ladders. Biochemistry 37: 6059–6064. 9558344 10.1021/bi972868g

[PETKOVICRNA047670C11] Danan M, Schwartz S, Edelheit S, Sorek R. 2012 Transcriptome-wide discovery of circular RNAs in Archaea. Nucleic Acids Res 40: 3131–3142. 22140119 10.1093/nar/gkr1009 PMC3326292

[PETKOVICRNA047670C12] DeYoung M, Siwkowski AM, Lian Y, Hampel A. 1995 Catalytic properties of hairpin ribozymes derived from Chicory yellow mottle virus and arabis mosaic virus satellite RNAs. Biochemistry 34: 15785–15791. 7495810 10.1021/bi00048a024

[PETKOVICRNA047670C13] Drude I, Vauléon S, Müller S. 2007 Twin ribozyme mediated removal of nucleotides from an internal RNA site. Biochem Biophys Res Commun 363: 24–29. 17825791 10.1016/j.bbrc.2007.08.135

[PETKOVICRNA047670C14] Drude I, Strahl A, Galla D, Müller O, Müller S. 2011 Design of hairpin ribozyme variants with improved activity for poorly processed substrates. FEBS J 278: 622–633. 21199369 10.1111/j.1742-4658.2010.07983.x

[PETKOVICRNA047670C15] Fedor MJ. 1999 Tertiary structure stabilization promotes hairpin ribozyme ligation. Biochemistry 38: 11040–11050. 10460159 10.1021/bi991069q

[PETKOVICRNA047670C16] Flamm C, Hofacker IL, Maurer-Stroh S, Stadler PF, Zehl M. 2001 Design of multistable RNA molecules. RNA 7: 254–265. 11233982 10.1017/s1355838201000863 PMC1370083

[PETKOVICRNA047670C17] Flores R, Grubb D, Elleuch A, Nohales MÁ, Delgado S, Gago S. 2011 Rolling-circle replication of viroids, viroid-like satellite RNAs and hepatitis delta virus: variations on a theme. RNA Biol 8: 200–206. 21358283 10.4161/rna.8.2.14238

[PETKOVICRNA047670C18] Grabowski PJ, Padgett RA, Sharp PA. 1984 Messenger RNA splicing in vitro: an excised intervening sequence and a potential intermediate. Cell 37: 415–427. 6722880 10.1016/0092-8674(84)90372-6

[PETKOVICRNA047670C19] Hammann C, Luptak A, Perreault J, de la Peña M. 2012 The ubiquitous hammerhead ribozyme. RNA 18: 871–885. 22454536 10.1261/rna.031401.111 PMC3334697

[PETKOVICRNA047670C20] Hampel A, Tritz R. 1989 RNA catalytic properties of the minimum (-)sTRSV sequence. Biochemistry 28: 4929–4933. 2765519 10.1021/bi00438a002

[PETKOVICRNA047670C21] Hansen TB, Wiklund ED, Bramsen JB, Villadsen SB, Statham AL, Clark SJ, Kjems J. 2011 miRNA-dependent gene silencing involving Ago2-mediated cleavage of a circular antisense RNA. EMBO J 30: 4414–4422. 21964070 10.1038/emboj.2011.359 PMC3230379

[PETKOVICRNA047670C23] Hegg LA, Fedor MJ. 1995 Kinetics and thermodynamics of intermolecular catalysis by hairpin ribozymes. Biochemistry 34: 15813–15828. 7495813 10.1021/bi00048a027

[PETKOVICRNA047670C24] Henn A, Medalia O, Shi SP, Steinberg M, Franceschi F, Sagi I. 2001 Visualization of unwinding activity of duplex RNA by DbpA, a DEAD box helicase, at single-molecule resolution by atomic force microscopy. Proc Natl Acad Sci 98: 5007–5012. 11296244 10.1073/pnas.071372498 PMC33154

[PETKOVICRNA047670C25] Ivanov SA, Vauleon S, Müller S. 2005 Efficient RNA ligation by reverse-joined hairpin ribozymes and engineering of twin ribozymes consisting of conventional and reverse-joined hairpin ribozyme units. FEBS J 272: 4464–4474. 16128815 10.1111/j.1742-4658.2005.04865.x

[PETKOVICRNA047670C26] Jeck WR, Sorrentino JA, Wang K, Slevin MK, Burd CE, Liu J, Marzluff WF, Sharpless NE. 2013 Circular RNAs are abundant, conserved, and associated with ALU repeats. RNA 19: 141–157. 23249747 10.1261/rna.035667.112 PMC3543092

[PETKOVICRNA047670C27] Liu S, Bokinsky G, Walter NG, Zhuang X. 2007 Dissecting the multistep reaction pathway of an RNA enzyme by single-molecule kinetic “fingerprinting”. Proc Natl Acad Sci 104: 12634–12639. 17496145 10.1073/pnas.0610597104 PMC1937518

[PETKOVICRNA047670C28] Liu Y, Cui H, Wang W, Li L, Wang Z, Yang S, Zhang X. 2013 Construction of circular miRNA sponges targeting miR-21 or miR-221 and demonstration of their excellent anticancer effects on malignant melanoma cells. Int J Biochem Cell Biol 45: 2643–2650. 24035906 10.1016/j.biocel.2013.09.003

[PETKOVICRNA047670C29] Lorenz R, Bernhart SH, Höner zu Siederdissen C, Tafer H, Flamm C, Stadler PF, Hofacker IL. 2011 ViennaRNA Package 2.0. Algorithms Mol Biol 6: 26. 22115189 10.1186/1748-7188-6-26 PMC3319429

[PETKOVICRNA047670C30] McCaskill JS. 1990 The equilibrium partition function and base pair binding probabilities for RNA secondary structure. Biopolymers 29: 1105–1119. 1695107 10.1002/bip.360290621

[PETKOVICRNA047670C31] Müller S, Appel B, Krellenberg T, Petkovic S. 2012 The many faces of the hairpin ribozyme: structural and functional variants of a small catalytic RNA. IUBMB Life 64: 36–47. 22131309 10.1002/iub.575

[PETKOVICRNA047670C32] Nahas MK, Wilson TJ, Hohng S, Jarvie K, Lilley DM, Ha T. 2004 Observation of internal cleavage and ligation reactions of a ribozyme. Nat Struct Biol 11: 1107–1113. 10.1038/nsmb842 15475966

[PETKOVICRNA047670C33] Ortinau S, Schmich J, Block S, Liedmann A, Jonas L, Weiss DG, Helm CA, Rolfs A, Frech MJ. 2010 Effect of 3D-scaffold formation on differentiation and survival in human neural progenitor cells. Biomed Eng Online 9: 70. 21070668 10.1186/1475-925X-9-70 PMC2996398

[PETKOVICRNA047670C34] Pasman Z, Been MD, Garcia-Blanco MA. 1996 Exon circularization in mammalian nuclear extracts. RNA 2: 603–610. 8718689 PMC1369399

[PETKOVICRNA047670C35] Petkovic S, Müller S. 2013 RNA self-processing: formation of cyclic species and concatemers from a small engineered RNA. FEBS Lett 587: 2435–2440. 23796421 10.1016/j.febslet.2013.06.013

[PETKOVICRNA047670C36] Pieper S, Vauleon S, Muller S. 2007 RNA self-processing towards changed topology and sequence oligomerization. Biol Chem 388: 743–746. 17570827 10.1515/BC.2007.067

[PETKOVICRNA047670C37] Rivetti C, Codeluppi S. 2001 Accurate length determination of DNA molecules visualized by atomic force microscopy: evidence for a partial B- to A-form transition on mica. Ultramicroscopy 87: 55–66. 11310542 10.1016/s0304-3991(00)00064-4

[PETKOVICRNA047670C38] Rivetti C, Guthold M, Bustamante C. 1996 Scanning force microscopy of DNA deposited onto mica: equilibration versus kinetic trapping studied by statistical polymer chain analysis. J Mol Biol 264: 919–932. 9000621 10.1006/jmbi.1996.0687

[PETKOVICRNA047670C39] Sigurdsson ST, Eckstein F. 1996 Isolation of oligoribonucleotides containing intramolecular cross-links. Anal Biochem 235: 241–242. 8833336 10.1006/abio.1996.0120

[PETKOVICRNA047670C40] Umekage S, Kikuchi Y. 2009 *In vitro* and *in vivo* production and purification of circular RNA aptamer. J Biotechnol 139: 265–272. 19138712 10.1016/j.jbiotec.2008.12.012

[PETKOVICRNA047670C41] Vauleon S, Ivanov SA, Gwiazda S, Muller S. 2005 Site-specific fluorescent and affinity labelling of RNA by using a small engineered twin ribozyme. ChemBiochem 6: 2158–2162. 16276501 10.1002/cbic.200500215

[PETKOVICRNA047670C42] Welz R, Bossmann K, Klug C, Schmidt C, Fritz HJ, Muller S. 2003 Site-directed alteration of RNA sequence mediated by an engineered twin ribozyme. Angew Chem Int Ed Engl 42: 2424–2427. 12783515 10.1002/anie.200250611

