# Supplementary material for: Sequence-controlled RNA self-processing: computational design, biochemical analysis, and visualization by AFM
Source: RNA. 2015 Jul;21(7):1249–60. doi: 10.1261/rna.047670.114 (PMC4478344; doi:10.1261/rna.047670.114)
Supplement: Supplemental Material [file supp_21_7_1249__index.html]

Sequence-controlled RNA self-processing: computational design, biochemical analysis, and visualization by AFM — Sequence-controlled RNA self-processing: computational design, biochemical analysis, and visualization by AFM — Supplemental Material 

# Sequence-controlled RNA self-processing: computational design, biochemical analysis, and visualization by AFM

## Supplemental Material

**Files in this Data Supplement:**

- Supp Material.pdf
