# Supplementary material for: Sequence-controlled RNA self-processing: computational design, biochemical analysis, and visualization by AFM
Source: RNA. 2015 Jul;21(7):1249–60. doi: 10.1261/rna.047670.114 (PMC4478344; doi:10.1261/rna.047670.114)
Supplement: Supplemental Material [file supp_047670.114_SuppMaterial.pdf]

# Supplemental Material

# Sequence-controlled RNA self-processing: computational design, biochemical analysis and visualization by AFM

Sonja Petkovic, Stefan Badelt, Stephan Block, Christoph Flamm, Mihaela Delcea, Ivo Hofacker, Sabine Müller

**Corresponding authors:**

Prof. Dr. Sabine Müller; Email: smueller@uni-greifswald.de

Prof. Dr. Ivo Hofacker; Email: ivo@tbi.univie.ac.at

Dr. Stephan Block; Email: stephan.block@chalmers.se

**Table of Contents**

Experimental S2

Design, preparation and analysis of an inactive cyclic dimer S4

Figure S1: Self-processing pathway of the designed RNAs S6

Figure S2: Models of the monomer and dimer cleavage cascades S7

Figure S3: Products of ribozyme reactions in preparative scale S8

Figure S4: 2D-Analyses of reference systems S9

Figure S5: AFM segment length histograms of reference and test systems S11

Figure S6: AFM contour histograms of references and test systems PBD1 and 4 S12

Figure S7: Comparison of AFM height and phase images of the l-83mer products S13

Estimation of activation energies (corresponding to Figure 7, main text) S14

Table S1: Sequences of self-processing RNAs S16

Table S2: Klenow primer sequences S17

References S18

# Experimental

### *General remarks and chemicals*

Deoxynucleotide triphosphates (dNTPs), nucleotide triphosphates (NTPs), Klenow buffer, DNase I, T7 RNA polymerase, Klenow fragment exo-, RiboLock™, RiboRuler™ low range RNA ladder and polynucleotide kinase were purchased from *Fermentas Company* (Schwerte, Germany); T4 RNA Ligase 2 (T4 RnL2) and the appropriate buffer was obtained from *New England Biolabs* (Frankfurt am Main, Germany). DNA primers were provided by *Biomers.net* (Ulm, Germany). RNase R including the required buffer was obtained from *epicenter* (Oldendorf, Germany). All chemicals and reagents were of analytical grade and filtered through a 0.2 µm polyvinyl difluoride membrane before use. Upon electrophoresis, all gels were stained for 5 to 10 min with ethidium bromide. Final concentration of ethidium bromide in 1xTBE was 0.5 µg/ml. All UV spectra were recorded on a NanoDrop ND 1000 spectrophotometer. Stained gels (agarose or polyacrylamide) were visualized using Chemi-Smart 2000 WL/LC 26M or VWR GenoView.

### *RNA preparation*

Klenow primers (for sequences see Supplemental Table S2) with 20 bp overlap (27 bp for the inactive dimer) were used in Klenow reactions with Klenow exo$^-$ polymerase following the manufacturer's protocol, and stopped by precipitation from ethanol at -20 °C overnight. DNA was isolated from native agarose gels (1.5%, EtBr stained). Product containing bands were cut out and DNA was isolated using QIA quick gel extraction kit (*Qiagen*, Venlo, The Netherlands). Since only one product was detectable after Klenow reaction, in later preparations the gel extraction step was skipped. Instead, after ethanol precipitation of the Klenow reaction product, the pellet was solved in 100 µl water, and 5 µl were used for subsequent *in vitro* transcription. RNAs were synthesized by *in vitro* transcription of double stranded DNA templates (1 µM concentration, or as mentioned above 5 µl of the Klenow product resolved in water after precipitation) with T7 RNA polymerase in the presence of the four ribonucleoside triphosphates (2 mM) and 1 U/µl RiboLock™ in 1x HEPES buffer (Na-HEPES 50 mM, $MgCl_2*6H_2O$ 12 mM, Spermidin 2 mM, pH = 7.5) in a total reaction volume of 50 µl for 3 h at 37 °C. DNA template was hydrolyzed adding 2 µl DNase I directly to the transcription mixture and left at 37 °C for additional 30-45 min. Final purification was achieved by electrophoresis on 15% denaturing polyacrylamide gels (for composition see subchapter *PAGE analysis* below), elution of the product-containing bands with sodium acetate (0.3 M, pH= 7, 3 times for at least two hours and overnight for the final elution step, shaking at approximately 500 rpm, at 10 °C) and precipitation with 250 vol.-% ethanol at -20 °C overnight.

***PAGE analysis***

For RNA species analysis or purification, denaturing (7 M urea) polyacrylamide gel electrophoresis (acrylamide: bisacrylamide 19:1 100 ml, ammonium persulfate 10 % w/v, 1 ml, *N,N,N′,N′*-Tetramethylethane-1,2-diamine 50 µl) was applied, using 1xTBE buffer as running buffer and stop mix (7 M urea, 50 mM EDTA, bromophenol blue and xylene cyanol each 5 vol-%) for sample loading. After mixing samples and/or RNA size standard with buffer, RNAs were denatured at 90 °C for 2 min and directly loaded onto the gel. Loading buffer for the RNA size standard was provided by *Fermentas* (Schwerte, Germany).

## Design, preparation and analysis of an inactive cyclic dimer

***Design of the inactive cyclic dimer***

Circular monomers have been successfully identified for CRZ-2 in our previous work (Petkovic and Muller 2013) however, the formation of circular dimers could not be shown. Since the size of cyclic RNAs is not assessable in PAA gels according to standard size markers, we designed an inactive circular dimer (CRZ*) as a reference. The cyclic reference dimer should be as similar as possible to the circular dimer of CRZ-2 and therefore help to identify this species in a PAA gel. However, due to its inactivity, the linear version of the reference dimer (produced by in vitro transcription) has to be ligated enzymatically to the desired cyclic product.

In particular, the design of an inactive circular dimer has to fulfill the following constraints with respect to the reference circular dimer (*CRZ*): *(i)* the secondary structure ensemble associated with the RNA has to be similar, *(ii)* the nucleotide content has to be equal, *(iii)* the sequence must not be symmetric, *(iv)* all conserved catalytic centers have to be destroyed and *(v)* a T7 promotor region is needed. Points *(i)* and *(ii)* shall insure a similar migration pattern on a polyacrylamide gel, whereas points *(iii)*, *(iv)* and *(v)* are necessary for experimental implementation. Asymmetry insures that only the defined 3'-terminal regions of Klenow primers overlap to obtain specific dsDNA of the desired length as template for RNA synthesis, and inactivity is necessary to avoid cleavage/ligation reactions after and during *in vitro* transcription. As a first step we preset the residual T7 RNA promotor sequence 5'-GGG AGA-3' as a non-mutable hexanucleotide at the 5'-end of the ribozyme. These bases will inevitably occur in the *in vitro* transcribed RNA due to usage of T7 RNA polymerase. Since this pre-processing step harms condition no. *(ii),* we mutated different helical regions to compensate for inequalities in the nucleotide content. Next, we randomly flipped base pairs within all helical regions (apart from the residual T7 RNA promotor region) and randomly shuffled the nucleotides from all loop regions to obtain loss of catalytic activity. With this approach we designed roughly 500 RNA species that fulfill conditions *(ii), (iii), (iv)* and *(v)*. To ensure similar folding behavior, the sequence should not only have the same ground state as the CRZ-2 dimer, also the whole structure ensemble should be similar. Therefore, we first selected for those sequences that have the smallest mean base pair distance within the equilibrium structure ensemble. This mean base pair distance (D) can be computed as

$$D(CRZ, CRZ^{*}) = \sum_{ij} P_{ij}^{CRZ}\left(1 - P_{ij}^{CRZ^{*}}\right) + P_{ij}^{CRZ^{*}}\left(1 - P_{ij}^{CRZ}\right) \quad [5]$$

With $P_{ij}^{CRZ}$ denoting the probability of a single base pair between position i and j for the molecule *CRZ*. From the top 20 designed molecules, we selected a sequence (shown in Table S2, Supporting Information) that has a comparable minimal free energy (MFE).

***Preparation of the inactive cyclic dimer by transcription priming with GMP***

To obtain the inactive linear dimer (*in-l-166mer)*, with 5'-terminal monophosphate, GMP was added to the NTP mix following the protocol of Harris and Christian for incorporation of guanosine monophosphorothioate.(Harris and Christian 1999) A 4.8:1 ratio of GMP:GTP was used, and the double stranded Klenow DNA, buffer, RiboLock™ and polymerase were added as described above. *In vitro* transcription was stopped after 3 hours at 37 °C, and double stranded DNA template was hydrolyzed using DNase I following manufacturer’s protocol. The reaction mixture was blended with 100 vol-% stop mix (7 M urea and 50 mM EDTA) and directly used for purification on a 15% denaturing polyacrylamide gel. After PAGE, elution of the desired RNA and ethanol precipitation as described above, RNAs were used for ligation. Enzymatic ligation in the double stranded region of the *in-l-166mer* to generate the cyclic species *in-c-166mer* was conducted using T4 RnL2 in a total reaction volume of 20 µl at 37 °C for 4 hrs following the suppliers protocol. RNA was purified using the RNA Clean & Concentrator™-5 kit (*ZymoResearch,* Freiburg, Germany*)* following the general protocol for total RNA purification. Elution of RNA was carried out with 50 µl desalted and purified millipore water. After addition of 50 µl stopmix, ligation products were analyzed on a 15% denaturing polyacrylamide gel.

***Digestion with RNase R***

10 µl of T4 RNA ligase 2 reaction mixture were used directly for hydrolysis using RNase R. $MgCl_2$ to a final concentration of 5 mM, RNase R buffer and water up to 17 µl were mixed and denatured at 90 °C for 5 min. The mixture was cooled down to 50 °C for 1 min before addition of 1 µl of an 1:1 freshly with water diluted RNase R solution. Hydrolysis occurred at 50 °C for 10 min. Reaction was stopped using an equal volume of stop mix, which is also used as loading buffer for electrophoresis, and the mixture was immediately frozen in liquid nitrogen. Reaction products were analyzed by electrophoresis through a 15% denaturing polyacrylamide gel.

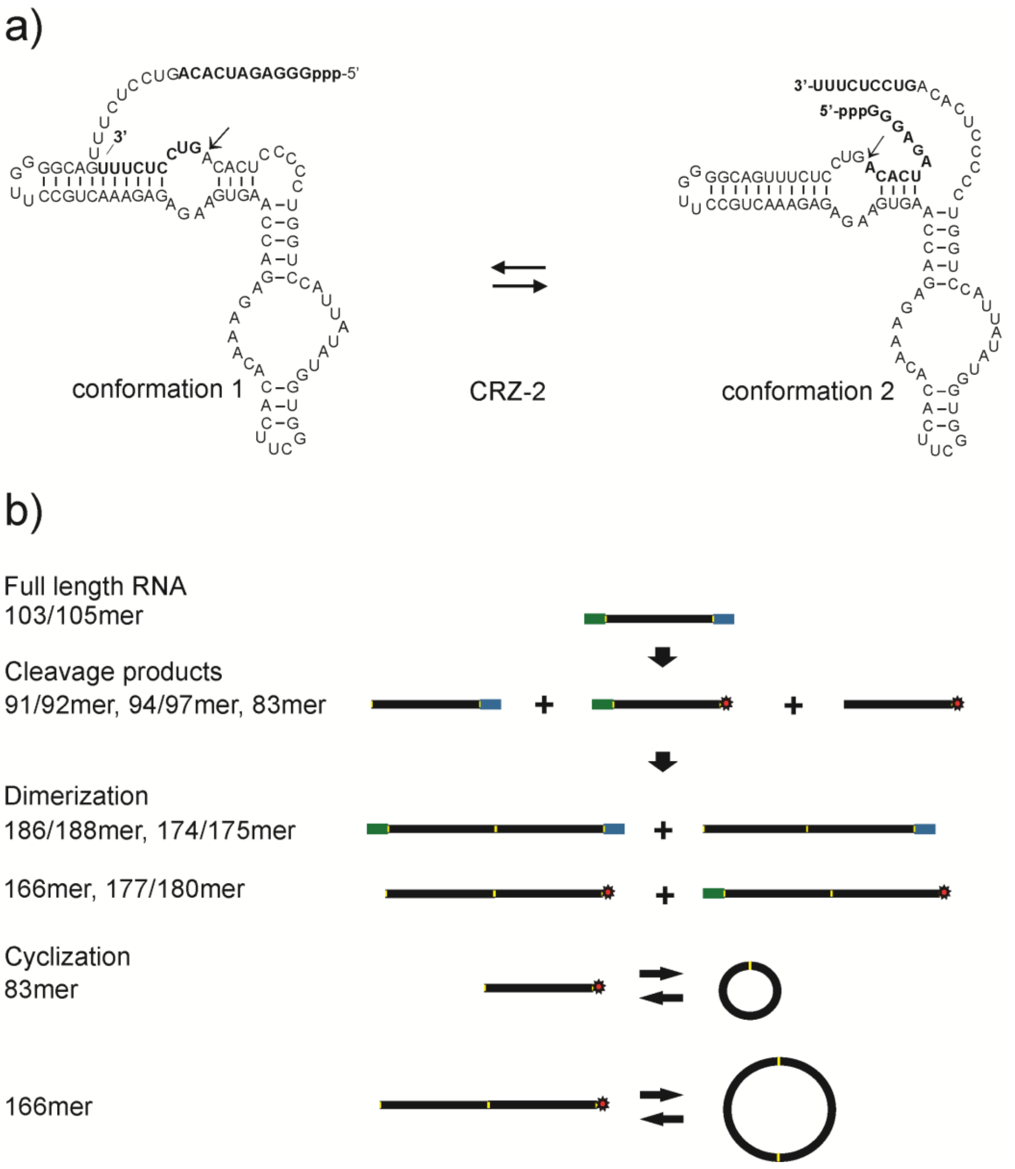


**Figure S1:** **a:** Two alternative cleavage-favoring conformations of the reference self-processing RNA CRZ-2, **b:** Schematic presentation of self-processing products.

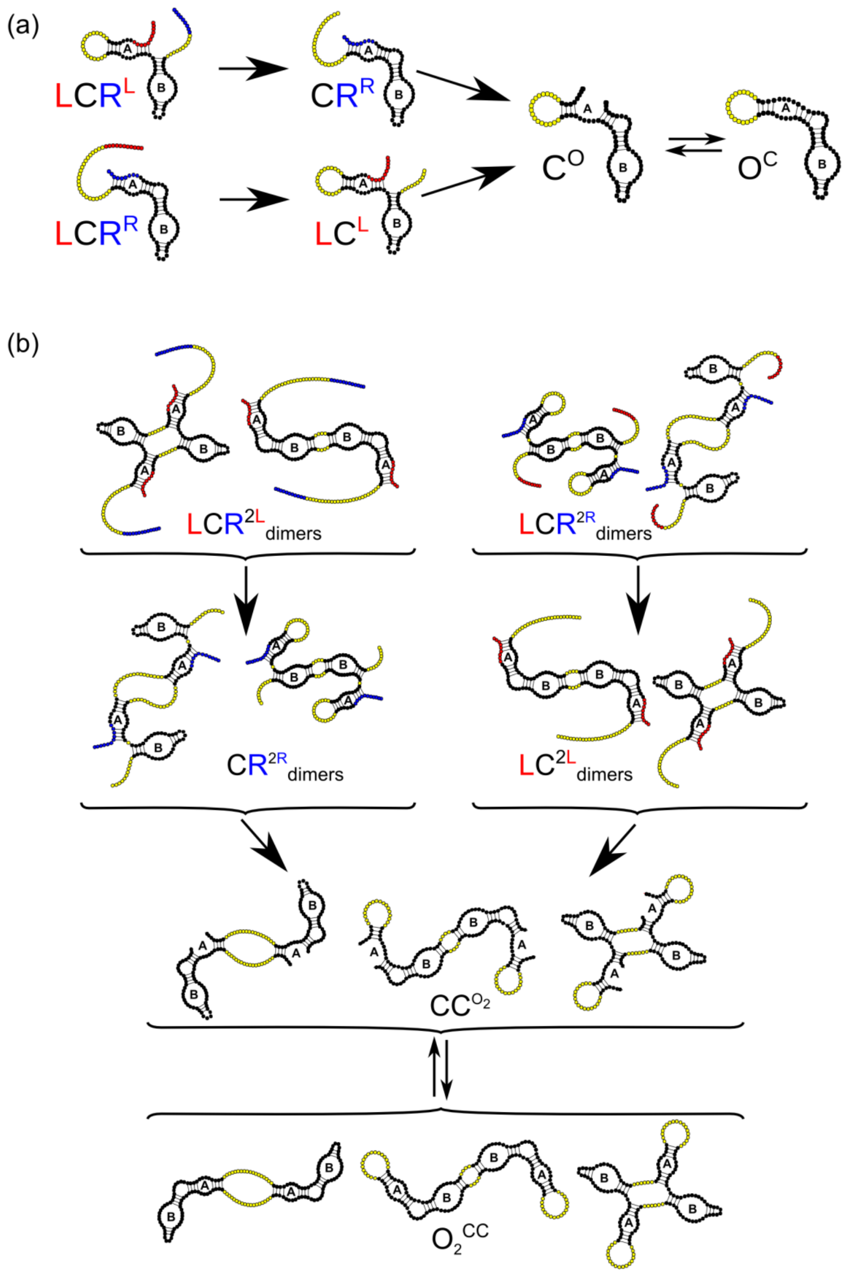


**Figure S2: Models of (a) the monomer and (b) dimer cleavage cascade**

Red and blue colored regions mark cleavable 5-’ and 3’-ends, respectively. A cleavage/ligation reaction can only occur when tertiary interactions between loop A and loop B are formed. In black we show structure constraints needed for such reactions, while yellow regions should be flexible without impairing catalytic activity. Importantly, every structure constraint defines a non-overlapping set of structures such that the probability of forming a reactive molecule can be computed from the sum of the constraint partition functions. RNA secondary structures were drawn using jViz (1).

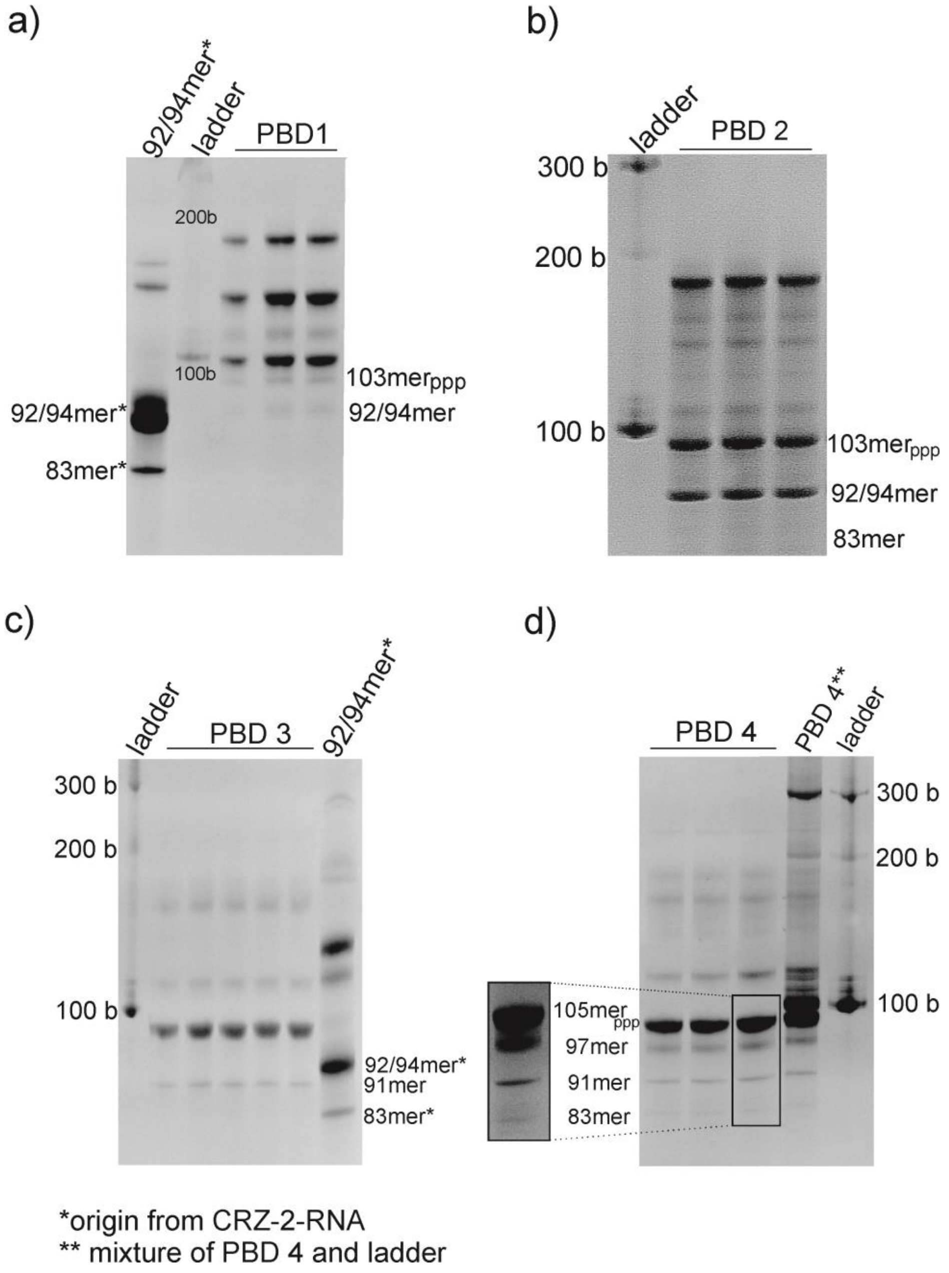


**Figure S3: Self-processing products of the four designed RNAs PBD1 to 4 analyzed with a 15% denaturing polyacrylamide gel (preparative scale).**

**a**: PBD1 self-processing products upon reaction at PBD1 starting concentration of 2.5 µM;
**b**: PBD2 self-processing products upon reaction at PBD2 starting concentration of 3 µM;
**c**: PBD3 self-processing products upon reaction at PBD3 starting concentration of 2 µM;
**d**: PBD4 self-processing products upon reaction at PBD4 starting concentration of 2 µM. For better visualization, the boxed area is shown slightly magnified and at higher contrast on the left.

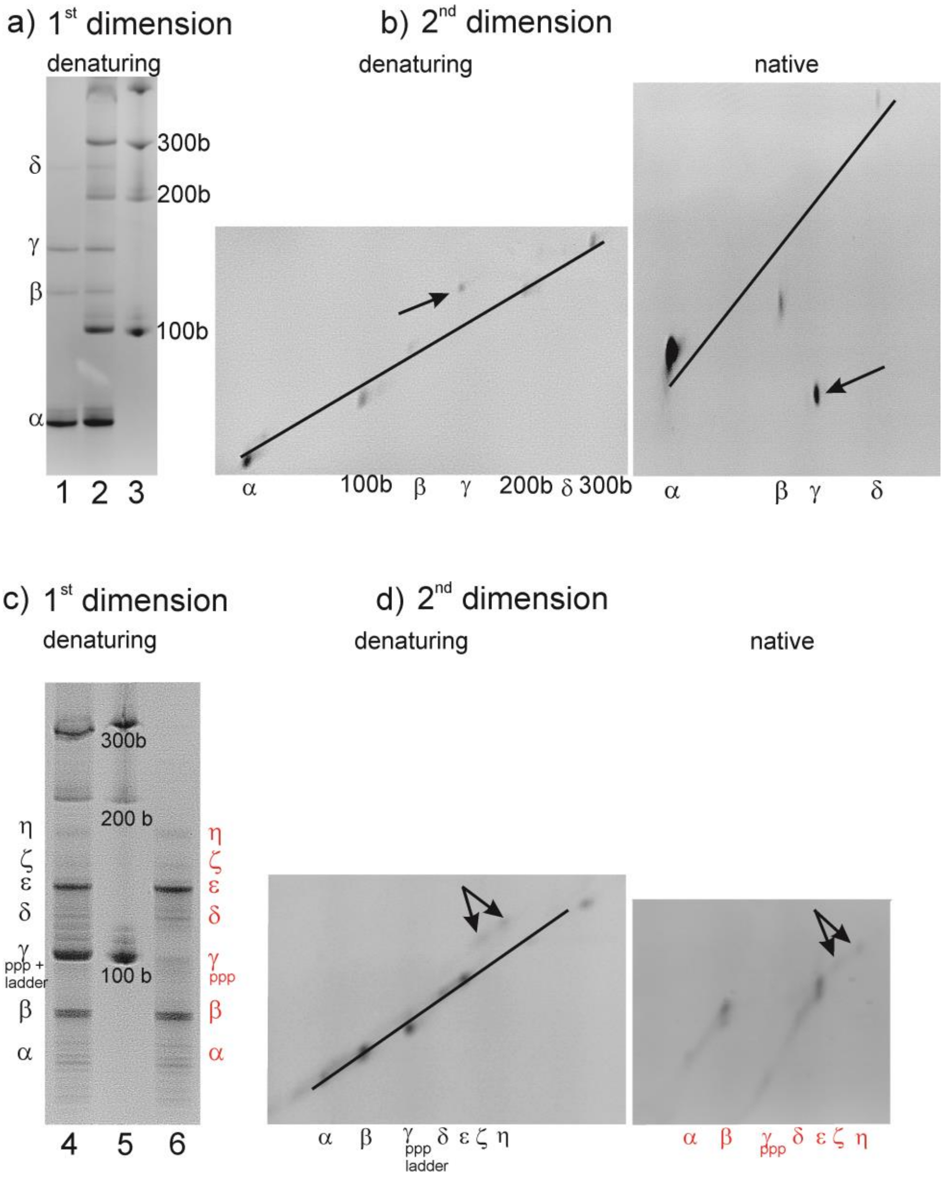


**Figure S4: Identification of cyclic RNA from l-83mer and from full-length CRZ-2 by 2D-PAGE.**

**a**: Self-processing of l-83mer analyzed on a 15% denaturing polyacrylamide gel. Lane 1: self-processing products denoted with Greek letters $\alpha$ to $\delta$. Lane 2: self-processing products mixed with linear RNA size standard. Lane 3: linear RNA size standard. **b**: Second-dimension denaturing (left, to improve resolution polyacrylamide concentration was increased to 17.5%) and native (right, 15%) polyacrylamide gels. Lane 1 of the gel shown in panel (**a**) was cut off and used as "starting slot" for the native gel in second dimension (**b**, right), lane 2 of the gel shown in panel (**a**) was equally used for the denaturing gel in second dimension (**b**, left). Species $\gamma$ (marked by an arrow) appears beyond the diagonal in both gels, implying its cyclic nature. **c**: Self-processing of full length CRZ-2 (103mer) analyzed on a 15% denaturing polyacrylamide gel. Lane 4: self-processing products denoted with Greek letters $\alpha$ to $\eta$, mixed with linear RNA size standard. Lane 5: linear RNA size standard. Lane 6: self-processing products denoted with Greek letters $\alpha$ to $\eta$. **d**: Second-dimension denaturing

(left, to improve resolution polyacrylamide concentration was increased to 17.5%) and native (right, 15%) polyacrylamide gels. Lane 4 of the gel shown in panel (**c**) was cut off and used as "starting slot" for the denaturing gel in second dimension (**d**, left), lane 6 of the gel shown in panel (**c**) was equally used for the native gel in second dimension (**d**, right). The two blurry spots $\zeta$ and $\eta$ marked by arrows in the denaturing gel in panel (**d**) might correspond to cyclic RNAs. However, the corresponding native gel on the right reveals that both RNAs do not migrate as fast as would be expected for cyclic species according to the analysis of l-83mer shown in panel (**b**).

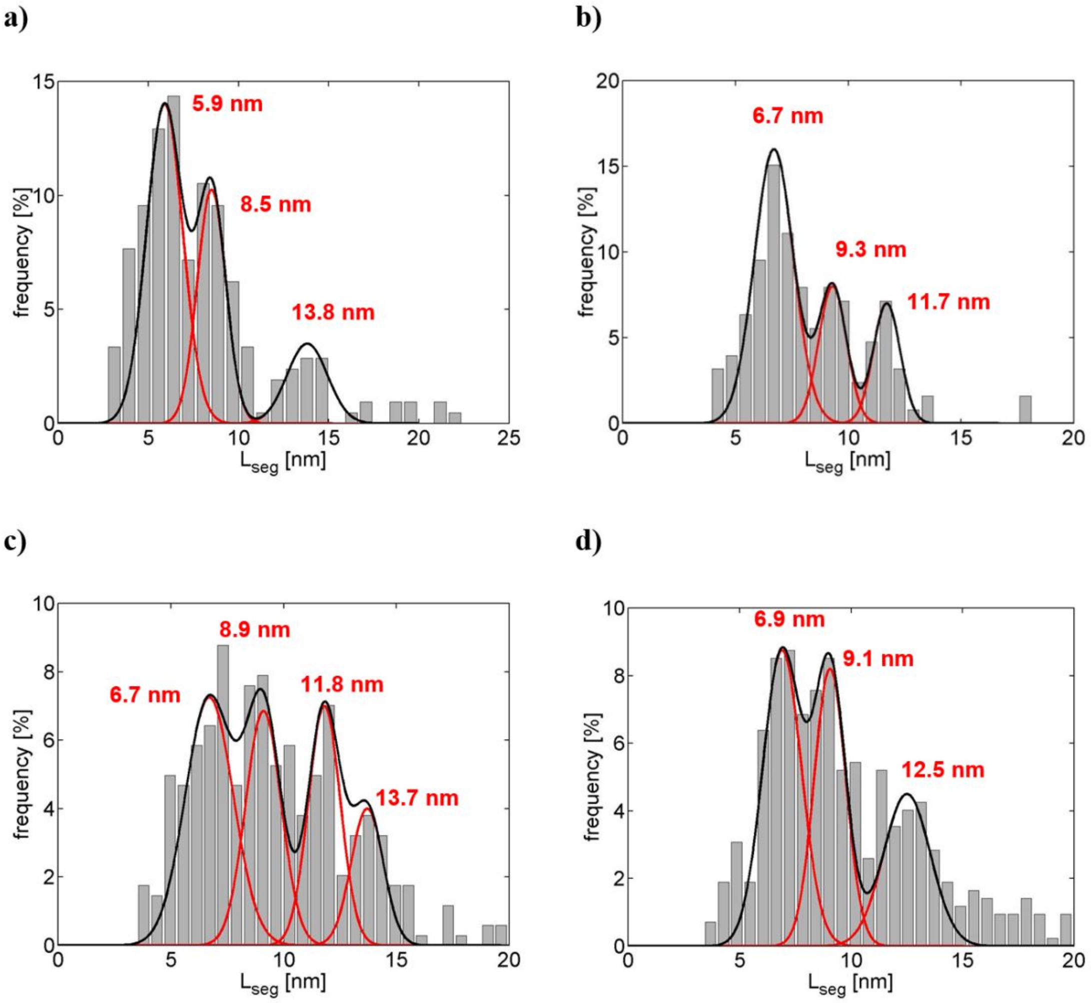


**Figure S5: RNA segment length histograms of references: (a) l-83mer, and (b) CRZ-2and test sequences (c) PBD1 and (d) PBD4**

In tapping mode AFM images the RNA chains typically consist of rod-like segments, which are connected via kinks (see Figs 5 and 6). From the AFM images, the length of these segments can be measured with nm accuracy, showing several well-resolvable peaks at (average ± standard deviation as averaged over the 4 different RNA sequences) 6.6±0.4 nm, 9.0±0.3 nm, 12.0±0.4 nm, and 13.8±0.1 nm. Assuming a double helical conformation of the rod-like segments and therefore a typical pitch of 0.3 nm per base pair, these peaks correspond to segments consisting of 22±1 bp, 30±1 bp, 40±1 bp, 46±0.3 bp. These histograms include the length of (**a**) 212, (**b**) 127, (**c**) 342 and (**d**) 423 segments.

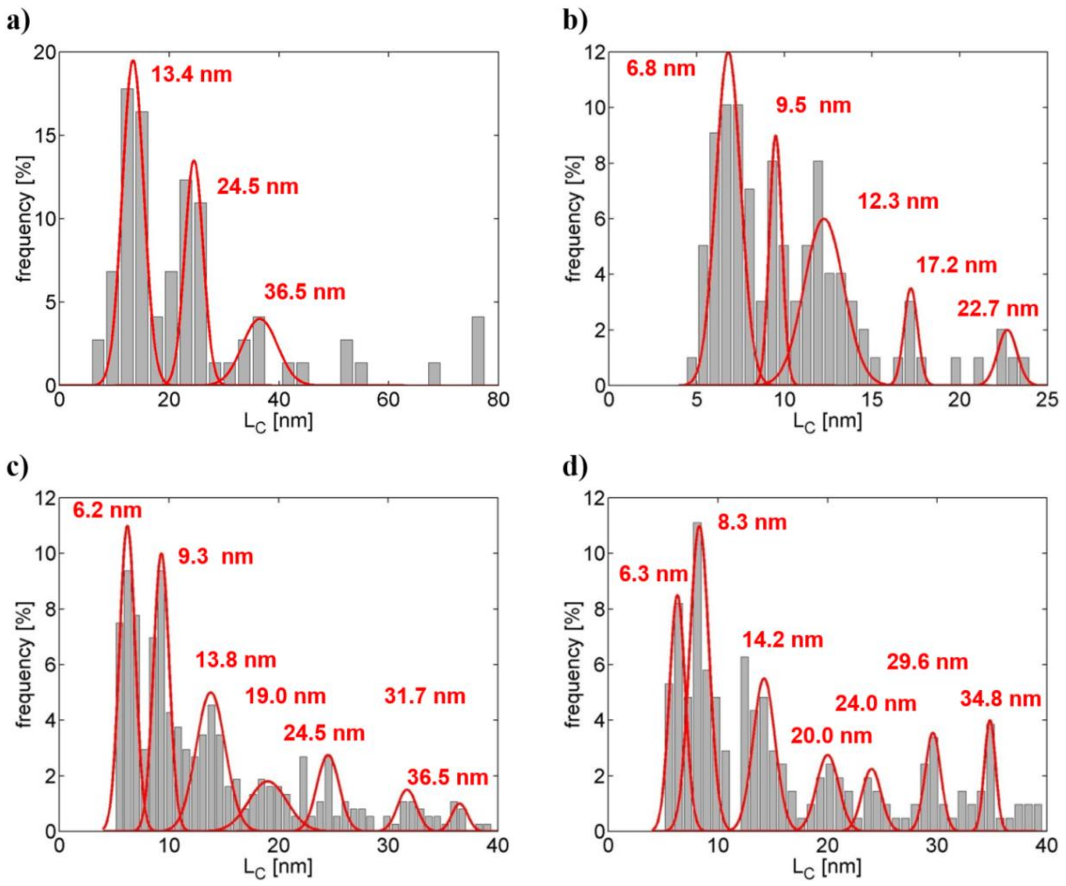


**Figure S6: Contour length histograms of RNA chains for (a) l-83mer, (b) CRZ-2, (c) PBD1, and (d) PBD4.**

For the linear 83mer, the histogram gives three peaks at 13.4, 24.5, and 36.5 nm. Assuming again a double helical conformation and therefore a typical pitch of 0.3 nm per base pair (= 0.15 nm per base), these peaks correspond to 89, 163 and 243 bases and can be identified as final cleavage products (83mer) and higher ligation products (dimer = 166mer; trimer = 249mer). (**b**-**d**) As the other RNA constructs additionally create intermediate cleavage products, their contour length histograms exhibit a more complicated peak structure. Generally, peaks are observed in all histograms at similar values allowing averaging the determined peak position over the three different RNA sequences. This yield the following values (average ± standard deviation of the respective peak position): 6.4±0.3 nm (first peak), 9.0±0.6 nm (second peak), 13.4±1.0 nm (third peak), 18.7±1.4 nm (fourth peak), 23.7±0.9 nm (fifth peak), and for PBD1 and PBD4 at 30.7±1.5 nm (sixth peak), and 35.7±1.2 nm (seventh peak). Note that the first two peaks coincide with the first two peaks of the segment length histograms in Figure S5. Hence, it is very likely that these peaks correspond to RNA chains, for which only one of the constituting segments was resolvable in the AFM image (*e.g.*, if two neighbouring segments enclose an angle of approximately 180° and therefore appear as a single segment in the measurement). These histograms include the contour length of (**a**) 73, (**b**) 100, (**c**) 218 and (**d**) 256 RNA chains.

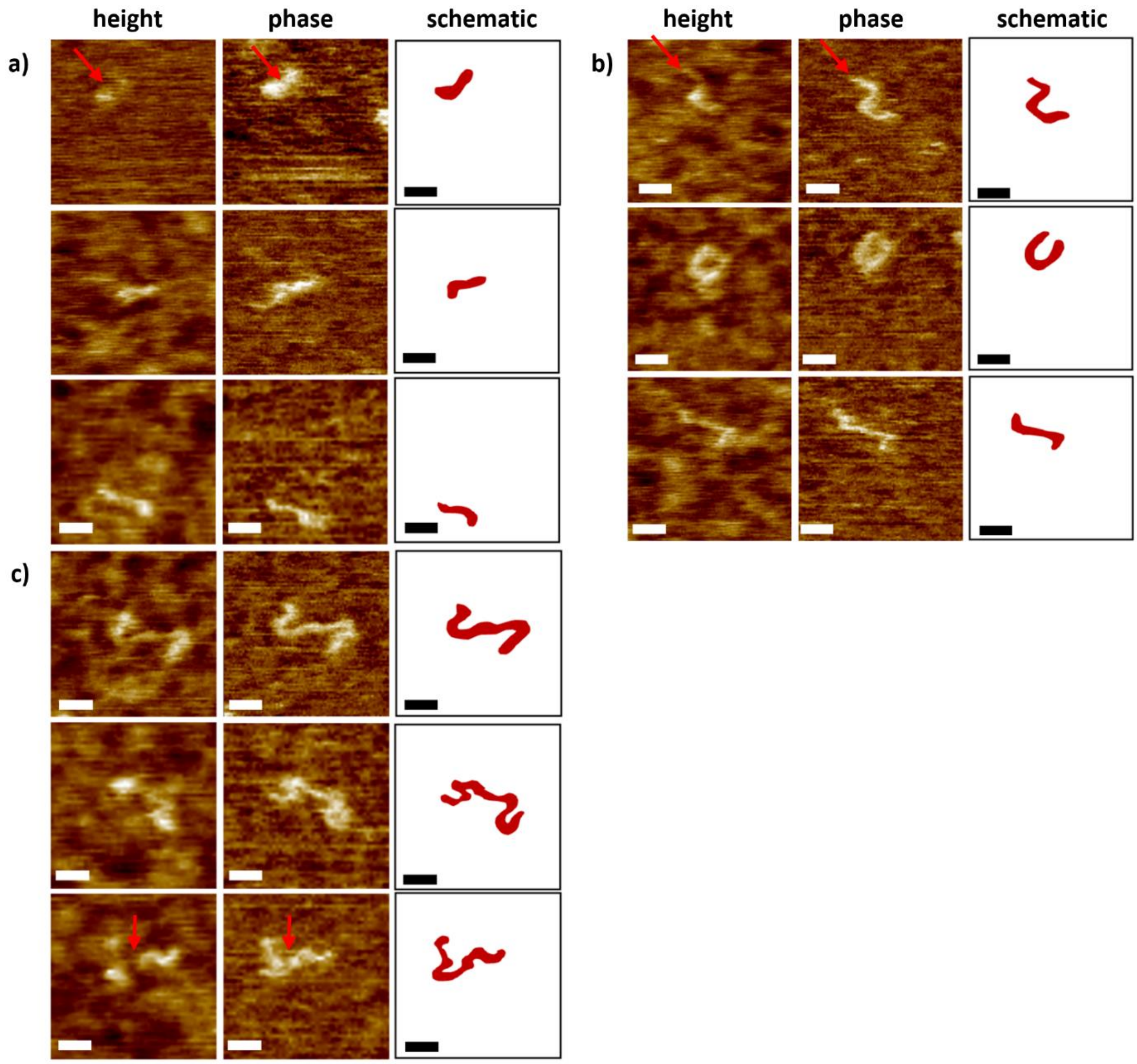


**Figure S7: Comparison of AFM height and phase images of the l-83mer products.**

Tapping mode (TM) AFM height images (height scale: 0.7 nm) and phase images (range: 0 – 30°) of the reaction products resulting from incubation of the l-83mer (isolated from CRZ-2 system) in cleavage/ligation buffer. Shown are 83mers (a), dimers (b) and trimers (c); scale bars correspond to 10 nm. For convenience, schematics have been included on the right side to help with the interpretation of the AFM images. For AFM analysis, samples were precipitated and resolved in 25 mM EDTA and 3.5 M urea (semi-denaturing conditions).

In most TM images, height and phase channels gave very similar results, i.e., they were in principle identical regarding RNA chain shape and regarding the obtained resolution. This holds for AFM tips with curvature radii down to approximately 4 nm (which was estimated from the resolution of the recorded AFM images) and applies for the majority of the measurements on CRZ-2, PDB-1, and PDB-1, so that for these ribozymes height images were given in the manuscript (Fig. 6). However, some AFM tips appeared to have a smaller

effective curvature radius, as indicated by a higher resolution in the phase channel and the tendency to deform/squeeze the ribozyme in the height image (compare the ribozyme structure at the positions indicated with red arrows in Fig. S7). The latter is understandable by the increased local pressure acting on the ribozyme if the interaction area is reduced (due to the reduction of the effective tip curvature radius). With these AFM tips the highest resolutions were achieved in this study, but with the sacrifice that sometimes parts of some ribozymes have been strongly deformed and are hard to see in the height channel, while the entire ribozyme is very well resolvable in the phase shift channel. As the l-83mer forms in principle only 3 products, such highly resolved images were obtained for all 3 products and therefore the phase images were given in the manuscript (Fig. 5), which enable the reader to get an impression of the ribozyme conformation with true nm resolution.

**Estimation of activation energies (Figure 7, main text)**

Figure 7 in the main paper shows a comprehensive view of the cleavage cascade for each of the experimentally tested ribozymes. We can distinguish three types of reaction steps *(i)* formation of reactive structures, *(ii)* dissociation of cleaved ends after ribozyme reaction, (iii) refolding of an unbound reaction product into a new reactive structure. Each of these steps is characterized by an activation free energy.

For *(i)* the Boltzmann probability of forming a reactive state is given by $\exp(-E_R/RT)/Z$, where $E_R$ denotes the energy of the reactive state and Z is the partition function (see Equation [1], main text). Thus, the corresponding activation energy is the difference between the free energy of the reactive state and the ensemble free energy $(-RT\ln(Z))$. This activation energy is optimized through cost function$\kappa_1$, energies to form reactive structures are therefore lower for all PBD molecules than for CRZ-2.

For *(ii)* and *(iii)* we approximate the best refolding path from the product conformation (reactive RNA dimer) to the next reactive species in the cascade. Finding the best refolding pathways is a computationally hard problem. The best direct refolding paths (i.e. paths of minimal length) can be estimated using the *findpath* heuristic (2). In order to get a better estimate of the energy barriers, we consider not only direct refolding paths but also detours via low-lying minima in the energy landscape. We computed low-lying minima of RNA landscapes with the program *barriers* (3) and selected the minimum free energy (MFE) conformation and up to three of the main alternative conformations. We then computed the direct refolding paths from the product conformation to each of these low-lying minima, from each low lying minimum the other and finally from each low-lying minimum to the reactive structure. The barriers along direct paths are computed as the difference between the worst energy along the refolding path the energy of the starting structure, the activation barriers *(ii)* and *(iii)* are selected such that the barrier of the total path is minimal. The dissociation barrier *(ii)* corresponds to the energy needed to dissociate the cleaved end, the refolding barrier *(iii)* describes the pathway from the unbound reaction product to the new reactive structure. The resulting values show that designed molecules often have to overcome higher dissociation barriers than CRZ-2.

**Table S1: Sequences of self-processing RNAs CRZ2 and PBD1- 4. Sequenes are shown with separated fragments resulting according to the cleavage sites. Further processing occurs by intra- and intermolecular ligation of the central 83mers. In addition, intermolecular ligation of central 83mers still containing either the 5'- or the 3'-tail can take place (comp. Figure S1).**

| | 5'-tail | central 83mer sequence | 3'-tail |
|---|---|---|---|
| **CRZ2** | GGGAGAUCACA-cp | HO-GUCCUCUUUGACGGGGUUCCGUCAAAGAGAGAAGUGAACCAGAGAAACACACUUCGGUGGUAUAUUACCUGGUCCCCCUCACA-cp | HO-GUCCUCUUU |
| **PBD1** | GGGAGAGCACA-cp | HO-GUCGGAGUUGCCGCGUUAGCGGCGGUUCUAGAAGUGCCCCGCAGAAACAGCCAUAUGGCGUAUAUUACGCGGGAAAAAGCACA-cp | HO-GUCGGAACC |
| **PBD2** | GGGAGAGAACA-cp | HO-GUCGGUGGUGCCCCGUAAGGGGCGUCGCCAGAAGUUCGGACCAGAAACAGCCAAAAGGCGUAUAUUACGGUCCAAAAAGAACA-cp | HO-GUCGGCGAC |
| **PBD3** | GGGAGHACA-cp | HO-GUCCGGUUUACCGCUAAUGCGGUGGGUCGAGAAGUCUGAGCGAGAAACACAGUAUACUGGUAUAUUACCGCUCCAUAAAGGCA-cp | HO-GUCCGGCACCAAAA |
| **PBD4** | GGGAGACA-cp | HO-GUCCGGUUUACCGCUAAUGCGGUGGGUCGAGAAGUCUGAGCGAGAAACACAGGACACUGGUAUAUUACCGCUCCAUAAAGGCA-cp | HO-GUCCGGCACCAAAA |

cp = 2', 3'-cyclic phosphate

**Table S2: Klenow primer sequences for generation of double-stranded DNA templates to be used for enzymatic synthesis of RNAs and of an inactive dimer**

| | Klenow primer 1 including T7 RNA promoter sequence (in italics) | Klenow primer 2 |
|---|---|---|
| PBD1 | 5’-*TAA TAC GAC TCA CTA TA* GGG AGA GCA CAG TCG GAG TTG CCG CGT TAG CGG CGG TTC TAG AAG TGC CCC GCA-3’ | 5’-GGT TGG CAC TGA GCT TTT TCC CGC GTA ATA TAC GCC ATA TGG CTG TTT CTG CGG GGC ACT TCT AGA ACC G-3’ |
| PBD2 | 5’-*TAA TAC GAC TCA CTA TA* GGG AGA GAA CAG TCG GTG GTG CCC CGT AAG GGG CGT CGC CAG AAG TTC GGA CCA G-3’ | 5’-TCG CCG ACT GTT CTT TTT GGA CCG TAA TAT ACG CCT TTT GGC TGT TTC TGG TCC GAA CTT CTG GCG ACG-3’ |
| PBD3 | 5’-*TAA TAC GAC TCA CTA TA* GGG AGA CAG TCC GGT TTA CCG CTA ATG CGG TGG GTC GAG AAG TCT GAG CGA GAA A-3’ | 5’-TTT TGG TGC CGG ACT GCC TTT ATG GAG CGG TAA TAT ACC AGT ATA CTG TGT TTC TCG CTC AGA CTT CTC GAC C-3’ |
| PBD4 | 5’-*TAA TAC GAC TCA CTA TA* GGG AGA CAG TCC GGT TTA CCG CTA ATG CGG TGG GTC GAG AAG TCT GAG CGA GAA ACA-3’ | 5’-TTT TGG TGC CGG ACT GCC TTT ATG GAG CGG TAA TAT ACC AGT GTC CTG TGT TTC TCG CTC AGA CTT CTC G-3’ |
| Inactive dimer | 5’-*TAA TAC GAC TCA CTA TA* GGG AGA GGT GTT TCA GAC TCG AGA ACC AGA GAA TGA CAC GTA TGT GCA GGA TTA ACT GGT AAA ACT CTC ACA GCT GAA ACA CCT CTT TCG G-3’ | 5’-GGT CTA CGA GGA TGG TCA GGA TAA GGT CGC AAG GTT GGT GGC AGC ACG CAT TAG GAC CTT GAC TTC GCT CAC AGA CCG AAA GAG GTG TTT CAG CTG TGA GAG-3’ |
| Full length RNA sequence of the inactive dimer | 5’-GGG AGA GGU GUU UCA GAC UCG AGA ACC AGA GAA UGA CAC GUA UGU GCA GGA UUA ACU GGU AAA ACU CUC ACA GCU GAA ACA CCU CUU UCG GUC UGU GAG CGA AGU CAA GGU CCU AAU GCG UGC UGC CAC CAA CCU UGC GAC CUU AUC CUG ACC AUC CUC GUA GACC-3’ | |

## References


1. Gan, H.H., Fera, D., Zorn, J., Shiffeldrim, N., Tang, M., Laserson, U., Kim, N. and Schlick, T. (2004) RAG: RNA-As-Graphs database--concepts, analysis, and features. *Bioinformatics*, **20**, 1285-1291.
2. Flamm, C., Hofacker, I.L., Maurer-Stroh, S., Stadler, P.F. and Zehl, M. (2001) Design of multistable RNA molecules. *RNA*, **7**, 254-265.
3. Flamm, C., Hofacker, I.L., Stadler, P.F. and Wolfinger, M.T. (2002) Barrier trees of degenerate landscapes. *Zeitschrift Fur Physikalische Chemie-International Journal of Research in Physical Chemistry & Chemical Physics*, **216**, 155-173.
